# Suboptimal Responses to Anti-VEGF in Retinal Neurovascular Diseases: Linking Aging and Alternative Angioinflammatory Pathways

**DOI:** 10.1167/iovs.67.5.4

**Published:** 2026-05-04

**Authors:** Somayeh Piroozmand, Hamid Latifi-Navid, Zahra-Soheila Soheili, Saman Hosseinkhani, Shahram Samiei, Amir Barzegar Behrooz, Hamid Ahmadieh, Andrea Leonardi, Saeid Ghavami, Nader Sheibani

**Affiliations:** 1 Department of Molecular Medicine, National Institute of Genetic Engineering and Biotechnology (NIGEB), Tehran, Iran; 2 School of Biological Sciences, Institute for Research in Fundamental Sciences (IPM), Tehran, Iran; 3 Departments of Ophthalmology and Visual Sciences, and Cell and Regenerative Biology, University of Wisconsin School of Medicine and Public Health, Madison, Wisconsin, United States; 4 Department of Biochemistry, Faculty of Biological Sciences, Tarbiat Modares University, Tehran, Iran; 5 Blood Transfusion Research Center, High Institute for Research and Education in Transfusion Medicine, Tehran, Iran; 6 Department of Human Anatomy and Cell Science, University of Manitoba College of Medicine, Winnipeg, Manitoba, Canada; 7 Ophthalmic Research Center, Research Institute for Ophthalmology and Vision Science, Shahid Beheshti University of Medical Sciences, Tehran, Iran; 8 Department of Neuroscience, Ophthalmology Unit, University of Padova, Padova, Italy; 9 Paul Albrechtsen Research Institute, CancerCare Manitoba, University of Manitoba, Winnipeg, Manitoba, Canada; 10 Biology of Breathing Theme, Children Hospital Research Institute of Manitoba, University of Manitoba, Winnipeg, Manitoba, Canada; 11 Akademia Śląska, Katowice, Poland

**Keywords:** anti-VEGF suboptimal responses, VEGF, systems biology, ocular diseases, angiogenesis, inflammation, aging

## Abstract

**Purpose:**

Vision-threatening ocular diseases are impacted by aging-associated molecular changes, including mitochondrial dysfunction, cellular senescence, and chronic inflammation. Anti-VEGF therapies targeting VEGF-A/VEGFR2 signaling remain the frontline standard of care, but many patients exhibit suboptimal or nondurable responses, often due to compensatory and/or compromised antiangiogenic and anti-inflammatory pathways. We aimed to elucidate shared mechanisms underlying treatment failure and disease progression.

**Methods:**

We applied an integrative systems biology framework that combined multiomics datasets, network-based machine learning, and disease-specific pathway mapping. A comprehensive literature review of conditions, including diabetic retinopathy, age-related macular degeneration, retinitis pigmentosa, glaucoma, and aging, identified 14 core genes consistently associated with angiogenesis, inflammation, and immune signaling. Multialgorithm centrality and enrichment analyses reconstructed disease-specific interaction networks, revealing consensus mechanistic axes. Integration of cell-type–specific single-cell RNA sequencing data from AMD–RPE clusters identified cluster-specific gene hubs and vertical signaling axes, leading to VEGF blockade failure.

**Results:**

EGFR, HSP90AA1, SIRT1, and STAT3 emerged as central resistance hubs linking angiogenesis and inflammatory processes. Pathway enrichment analyses revealed 21 conserved core signaling cascades, grouped into six functional categories, with AGE–RAGE, PI3K–Akt, HIF-1, MAPK, and chemokine pathways playing central roles. A MiRGD-based peptide nanocomplex delivering htsFLT01 achieved efficient RPE transfection and controlled gene activation under basal conditions.

**Conclusions:**

This systems-level framework clarifies mechanisms of VEGF blockade resistance and provides a rational basis for next-generation, combinatorial therapeutic strategies requiring validation in disease-relevant models.

Age-related macular degeneration (AMD) is a devastating retinal disorder and the leading cause of irreversible vision loss in individuals older than 55 years in industrialized countries. Global projections suggest that nearly 288 million people will be affected by 2040, imposing an enormous health and socioeconomic burden worldwide.^1^^,^^2^ In its neovascular form (nAMD), aberrant blood vessel growth beneath the macula results in fluid exudation, hemorrhage, and photoreceptor degeneration, culminating in central vision loss and impairment in daily activities such as reading and facial recognition.^3^^–^^5^

AMD pathogenesis is multifactorial, involving oxidative stress, chronic inflammation, autophagy dysfunction, dysregulated lipid metabolism, complement activation, and aging-associated cellular and molecular alterations.^6^^,^^7^ A hallmark of nAMD is pathologic angiogenesis, largely driven through enhanced vascular endothelial growth factor (VEGF) production and signaling pathways. VEGF binding to its principal receptor, VEGFR2, triggers endothelial cell proliferation and migration, as well as neovascularization, key features of nAMD progression.^8^^–^^11^ Consequently, anti-VEGF agents have become standard-of-care therapies aimed at trapping VEGF and interfering with its VEGFR2 interactions to mitigate vascular growth.^12^^–^^15^

Despite remarkable clinical success, anti-VEGF therapies face critical limitations. A considerable subset of patients exhibits inadequate or suboptimal therapeutic responses to anti-VEGF treatments, highlighting the involvement of compensatory and/or compromised angioinflammatory pathways bypassing VEGF–VEGFR2 signaling.^10^^,^^16^^,^^17^ Additionally, the high frequency of intravitreal injections imposes logistical and financial burdens and may accelerate retinal atrophy.^18^ Gene therapy–based approaches are emerging as promising strategies for sustained VEGF suppression with fewer interventions.^19^ However, both single-target and multitarget approaches are hindered by incomplete knowledge of the complex signaling circuits linking VEGF or other proangiogenic and inflammatory factors to downstream parallel signaling pathways.

The complexity of AMD is further intensified by its strong association with aging. Other retinal disorders, such as diabetic retinopathy (DR) and retinal vein occlusion, also follow age-dependent trajectories, implicating cellular senescence, mitochondrial dysfunction, telomere attrition, and dysregulated nutrient signaling in their pathophysiology.^20^^–^^25^ Aging not only contributes to disease development but also modifies tissue responsiveness to therapeutic agents, including antiangiogenic and anti-inflammatory drugs. Therefore, integration of aging biology with angiogenesis and inflammation research is critical for developing effective treatments.

This interplay extends into broader networks, where angiogenesis interfaces with inflammation, immune activation, vascular remodeling, metabolic regulation, cellular stress, and developmental signaling.^26^^–^^39^ These interconnected pathways may underlie suboptimal response to current therapies and offer new, broadly applicable molecular targets across retinal neurovascular degenerative conditions. A deeper understanding of these interconnected mechanisms may reduce the need for condition-specific drug design and help overcome one-size-fits-all limitations.

Interactome-based analyses can predict molecular commonalities between phenotypically related diseases, even when they do not share primary disease genes.^40^ This supports the use of systems-level comparisons across disease contexts (e.g., glaucoma [GLAU]/retinitis pigmentosa [RP] vs. AMD/DR) to identify shared and distinct network modules that may inform cross-disease hypotheses.

Therefore, to design a systematic study evaluating antiangiogenic molecules—with a focus on their mechanisms of action, benefits, limitations, and the identification of key hub genes that may contribute to reduced therapeutic efficacy—a comprehensive, multifaceted approach is essential. This must encompass careful selection of the delivery vehicle for gene therapy (MiRGD), the specific antiangiogenic molecule (htsFLT01), and the critical hub genes involved in mediating suboptimal responses to the molecule's effects (anti-VEGF responsiveness).

It is precisely at this juncture that a logical and mechanistic connection emerges between the htsFLT01/MiRGD nanocomplex and anti-VEGF responsiveness. Specifically, the nanocomplex either demonstrates the capacity to induce significant alterations in key genes implicated in suboptimal responses to the antiangiogenic molecule or lacks this capability. In the former scenario, this attribute would constitute a positive feature of the nanocomplex, providing a stronger foundation for deeper investigation and improved understanding of the antiangiogenic mechanisms exerted by the htsFlT01 molecule. Conversely, in the latter case, the findings could yield novel insights into the reconfiguration and redesign of antiangiogenic molecules, enabling the development of agents capable of simultaneously inhibiting and neutralizing multiple key proangiogenic proteins involved in the emergence of suboptimal responses to antiangiogenic therapies.

The htsFLT01 is a chimeric protein and has been optimized for enhanced stability and flexibility of its ligand (VEGF)–binding domain while reducing antigenicity. The in vivo antiangiogenic role of this protein has been previously demonstrated through intravitreal injection of rAAV2–htsFLT01 particles and MiRGD/htsFLT01 complex into the ocular space of neonatal mice.^19^ The MiRGD peptide-based carrier was selected as the nonviral delivery vehicle for the htsFLT01-encoding plasmid due to several key advantages. First, MiRGD enables integrin-targeted gene transfer specifically to retinal pigment epithelium (RPE) cells, the primary source of VEGF production in the outer retina in neovascular eye diseases. Second, it forms stable electrostatic nanocomplexes that protect plasmid DNA from nuclease degradation while promoting efficient cellular uptake and transfection. Third, this platform has already been validated with htsFLT01 in our previous work, where the MiRGD/htsFLT01 nanocomplex demonstrated antiangiogenic effects both in vitro—through inhibition of human umbilical vein endothelial cells (HUVEC) tube formation—and in vivo in models of physiological neovascularization. Collectively, these attributes position MiRGD as a promising, biocompatible, and targeted nonviral system for ocular anti-VEGF gene therapy.^41^

Here, we utilized a systems biology approach coupled with machine learning to potentially identify alternative angiogenic and aging-related pathways and to reveal common regulatory hubs among AMD, DR, GLAU, and RP. Our goal was to determine why some patients have a suboptimal response to the VEGF blockade and how aging may shape disease trajectory at the molecular level. We initially included glaucoma and retinitis pigmentosa in our analysis as part of a systems-level mapping of aging and inflammatory mechanisms across diverse retinal pathologies. By comparing a primarily VEGF-driven neovascular disease (AMD/DR) with neurodegenerative retinal conditions (GLAU/RP), we aimed to identify common or divergent molecular pathways—for example, chronic “inflammaging” processes—that might be shared across retinal diseases even if their proximal drivers or therapies differ. This broad approach was intended to generate new hypotheses about alternative angioinflammatory pathways that could contribute to anti-VEGF suboptimal responses.

Furthermore, we evaluated the functional impact of htsFLT01, a chimeric VEGF-neutralizing protein delivered via MiRGD-mediated transfection, on the expression of angiogenesis- and aging-related genes in human RPE cells.^19^^,^^41^^–^^44^ Because RPE cells are the primary regulators of retinal VEGF production and paracrine signaling to endothelial cells, we focused our mechanistic analyses on RPE rather than endothelial cells.

To our knowledge, this is the first study to integrate AMD, DR, RP, and glaucoma data with aging pathways in a unified analysis. This systems-level strategy—combined with multilayer machine learning and subsequent experimental nanodelivery validation—represents a novel and innovative approach in ophthalmic research. Figure 1 provides an overview of this integrated strategy, illustrating the transition from VEGF/VEGFR2-mediated angiogenesis to its suppression by the htsFLT01/MiRGD nanocomplex, while highlighting the role of compensatory pathways in anti-VEGF suboptimal response as revealed by systems biology and network-based machine learning (Fig. 1; Supplementary Fig. S1).

**Figure 1.**
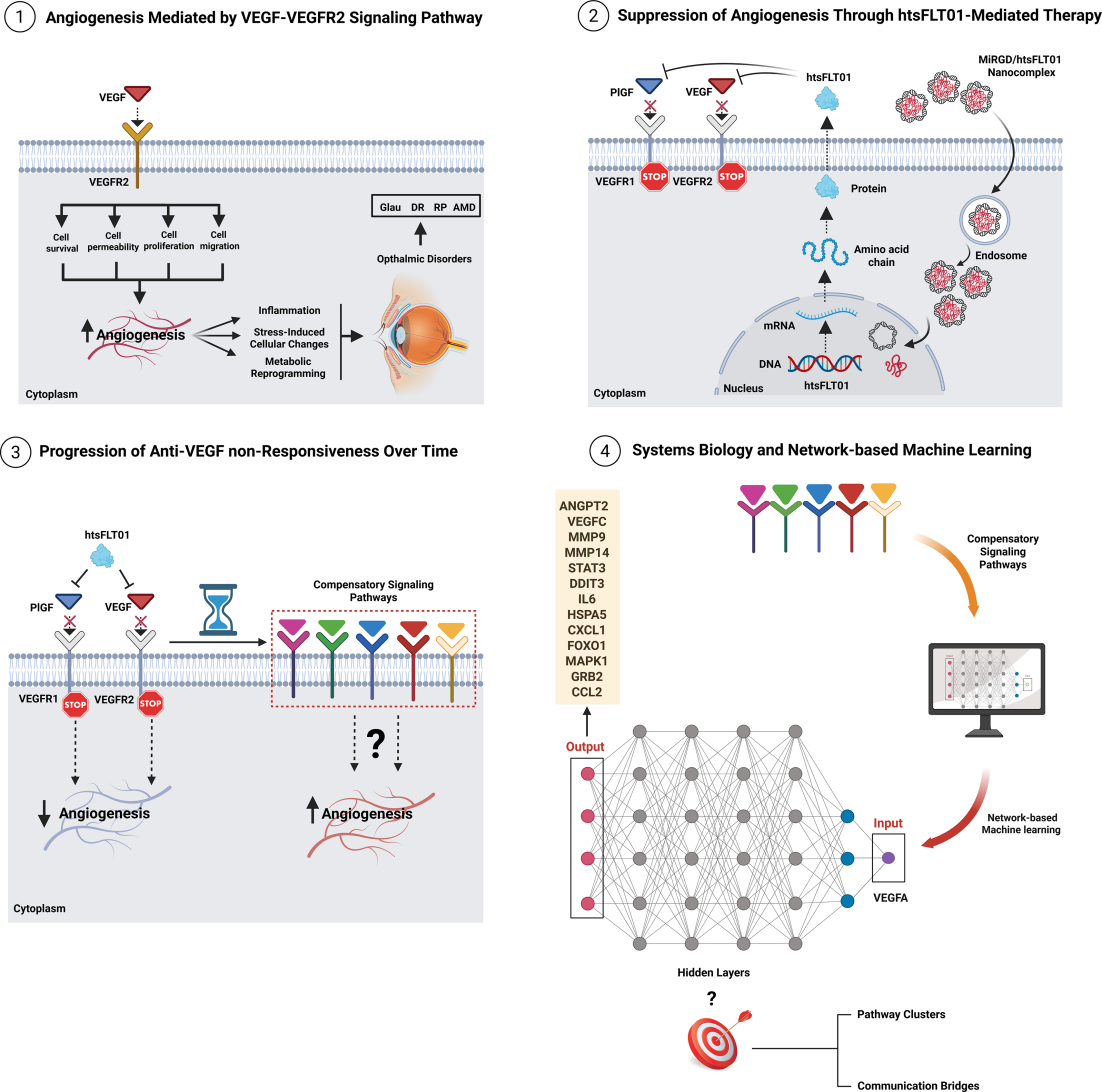
**Panel 1:** Angiogenesis Mediated through VEGF/VEGFR2 Signaling Pathway. When VEGF binds to its receptor VEGFR2, it triggers signaling pathways that promote cell migration, proliferation, and increased vascular permeability; key steps in angiogenesis. However, when combined with inflammation, oxidative stress-induced cellular changes, and abnormal metabolic alterations, the VEGF-VEGFR2 pathway can contribute to the development of serious ophthalmic disorders, including AMD, DR, RP, and GLAU. **Panel 2:** Mitigation of Angiogenesis Through htsFLT01-Mediated Therapy. The htsFLT01/MiRGD nanocomplex, via MiRGD peptide motifs, attaches to cells, escapes from the endosome, and is transported to the nucleus. htsFLT01 is then expressed and secreted, binding to the PlGF and VEGF. This binding competitively inhibits ligand-receptor interactions with VEGFR1 and VEGFR2. Ultimately, pro-angiogenic signaling pathways are effectively suppressed. **Panel 3:** Anti-VEGF Suboptimal Response. Despite the considerable antiangiogenic efficacy of htsFLT01 and other agents that block the primary VEGF-VEGFR2 axis, a potential for suboptimal response to these treatments exists. This occurs due to compensatory and/or compromised anti-angiogenic and anti-inflammatory pathways that facilitate the development of new vasculature despite the blockage of the implicated primary pathway. Examining the function of these compensatory and/or compromised anti-angiogenic and anti-inflammatory pathways in the emergence of anti-VEGF suboptimal response is a matter that requires attention. **Panel 4:** Systems Biology and Network-based Machine Learning. The examination of the hidden layers in the information transfer from VEGF (input data) to output data within compensatory and/or compromised anti-angiogenic and anti-inflammatory pathways resulted in the identification of a black box that may contribute to the development of anti-VEGF suboptimal response involving pathway clusters and communication bridges. The schematic was designed by BioRender (https://BioRender.com/q2kawlt).

## Materials and Methods

### Bioinformatics Studies

#### Data Source 1 (Related to Pathways)

**Figure 2.**
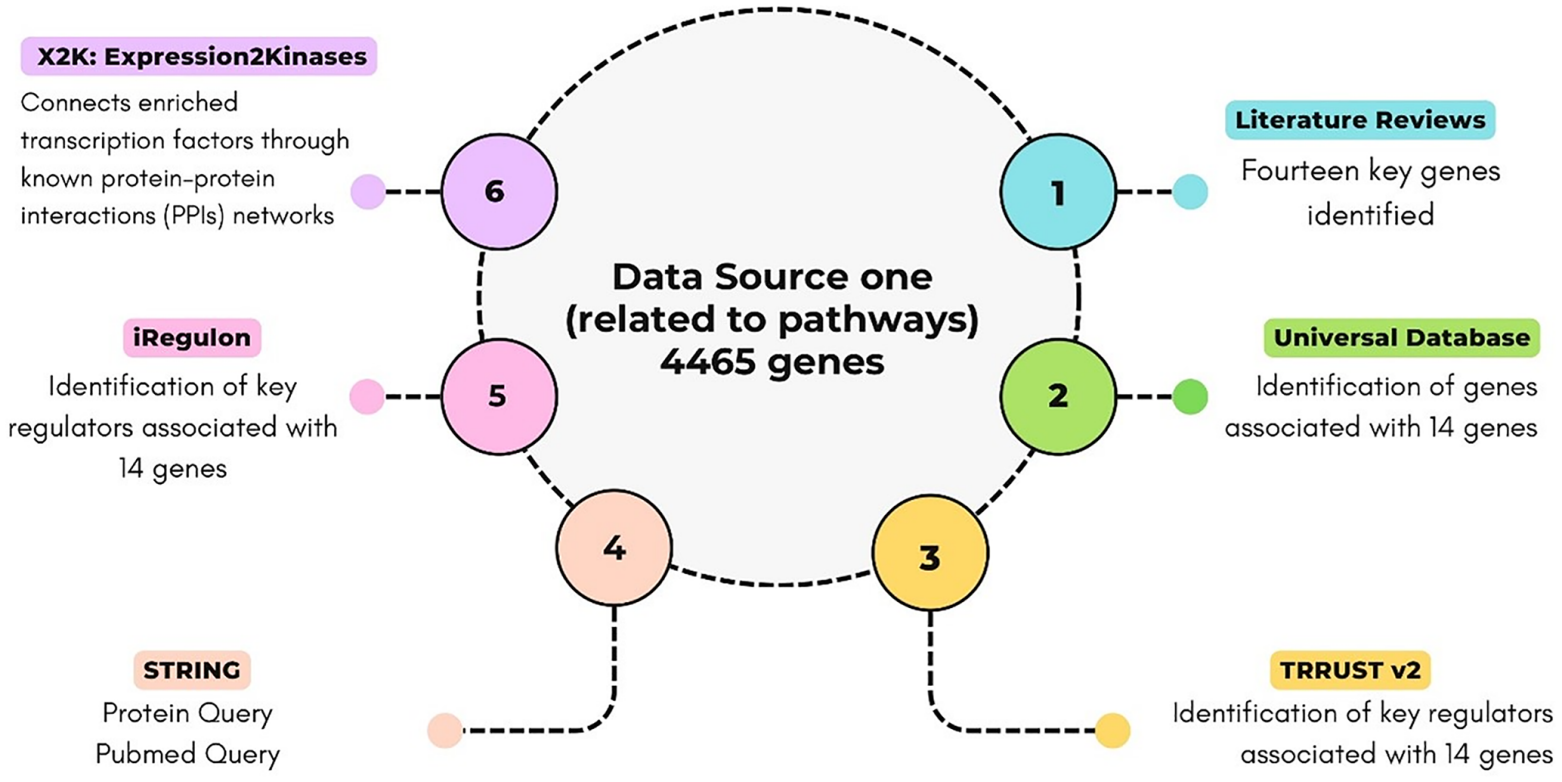
Integrated data sources and analytical platforms used to identify key regulatory genes. A total of 4465 pathway-related genes were analyzed using complementary resources. Literature reviews (1) and universal databases (2) provided 14 core genes. TRRUST v2 (3) and iRegulon (5) identified transcriptional regulators associated with these genes, while STRING (4) enabled protein–protein and PubMed queries. Finally, X2K (6) connected enriched transcription factors through protein–protein interaction networks. Together, these resources formed a multilayered framework for uncovering candidate regulators in the study.

In this study, we identified proteins involved in shared pathways across AMD, DR, GLAU, and RP by reviewing relevant studies using angiogenesis-, inflammation-, and immune response–related keywords. Fourteen core genes were identified from published studies and existing knowledge (Table 1). To explore functional associations, related genes were retrieved using TRRUST v2 (www.grnpedia.org/trrust),^45^ STRING (https://string-db.org/) (confidence score = 0.9),^46^ iRegulon (http://iregulon.aertslab.org/),^47^ and X2K (https://maayanlab.cloud/X2K/).^48^ STRING's protein and PubMed queries yielded a total of 4465 genes. The full workflow is shown in Figure 2 and Supplementary Table S1.

#### Data Source 2 (Related to Disease and Aging)

**Figure 3.**
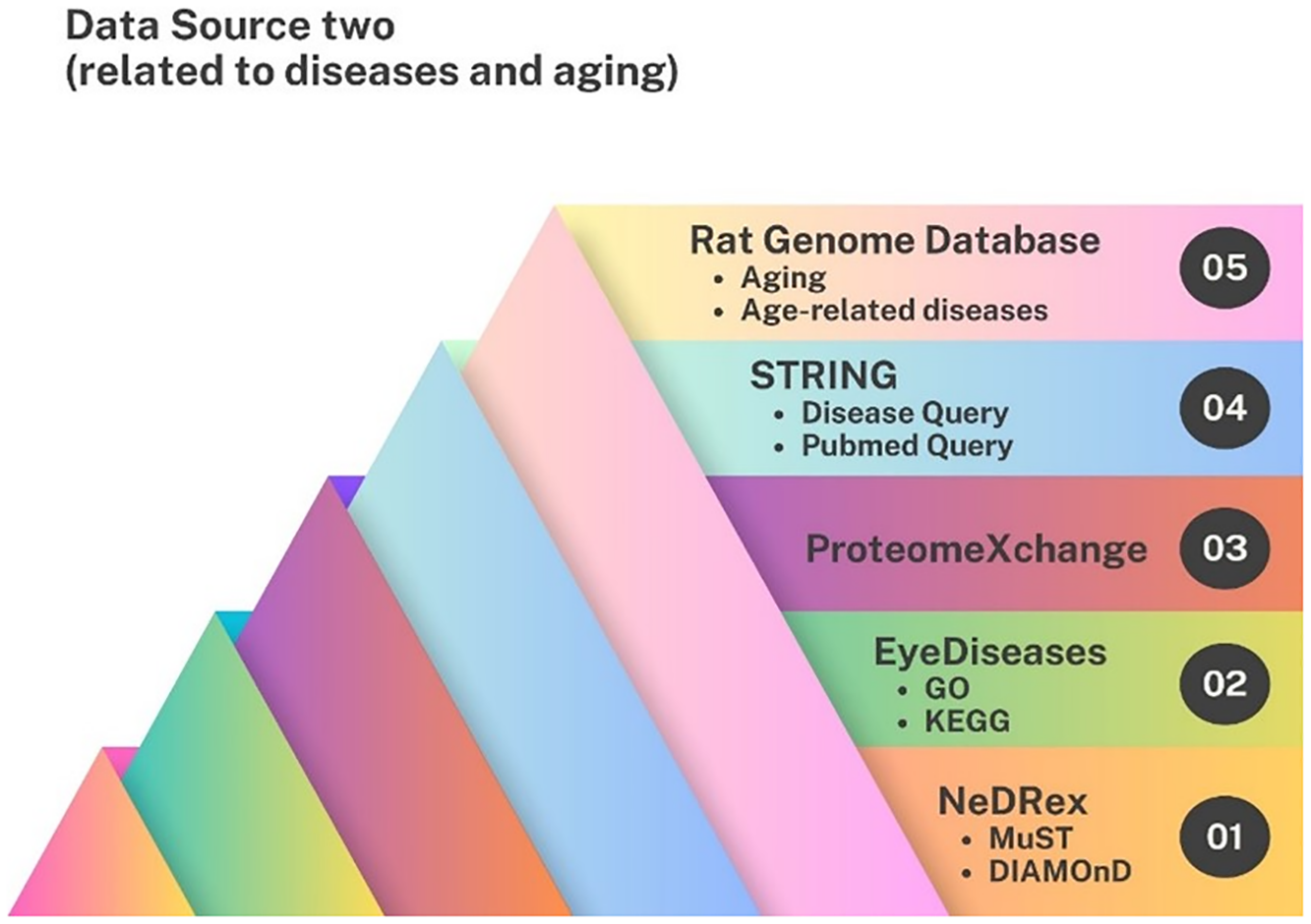
Data sources related to diseases and aging used for gene and pathway identification. To explore disease- and age-related regulators, five complementary resources were utilized. NeDRex (1) applied MuST and DIAMOnD algorithms to identify network-based disease associations. EyeDiseases (2) integrated GO and KEGG annotations, while ProteomeXchange (3) provided proteomics datasets. STRING (4) enabled disease-specific and PubMed queries, and the Rat Genome Database (5) offered curated information on aging and age-related diseases. Together, these databases formed a multidimensional framework for linking aging and disease processes to regulatory gene networks.

Genes associated with the four diseases were identified using EyeDiseases (https://eyediseases.bio-data.cn/),^49^ ProteomeXchange (https://www.proteomexchange.org/),^50^ STRING (https://string-db.org/) (confidence score = 0.9),^46^ RGD (https://rgd.mcw.edu/),^51^ and the NeDRex (https://nedrex.net/) plugin in Cytoscape (https://cytoscape.org/). NeDRex utilized MuST and DIAMOnD algorithms.^52^ From RGD (https://rgd.mcw.edu/), data on chemicals, phenotypes, biological processes, pathways, and diseases were examined; from EyeDiseases (https://eyediseases.bio-data.cn/), gene ontology and Kyoto Encyclopedia of Genes and Genomes (KEGG) (https://www.genome.jp/kegg/) terms were explored; and from STRING (https://string-db.org/) (confidence score = 0.9), disease and PubMed queries were analyzed. This yielded 581 genes for AMD (Supplementary Table S2), 1087 for DR (Supplementary Table S3), 1290 for GLAU (Supplementary Table S4), 1366 for RP (Supplementary Table S5), and 1391 for aging (Supplementary Table S6). The workflow is shown in Figure 3.

#### Network Reconstruction and Centrality Analysis to Identify Key Genes

**Figure 4.**
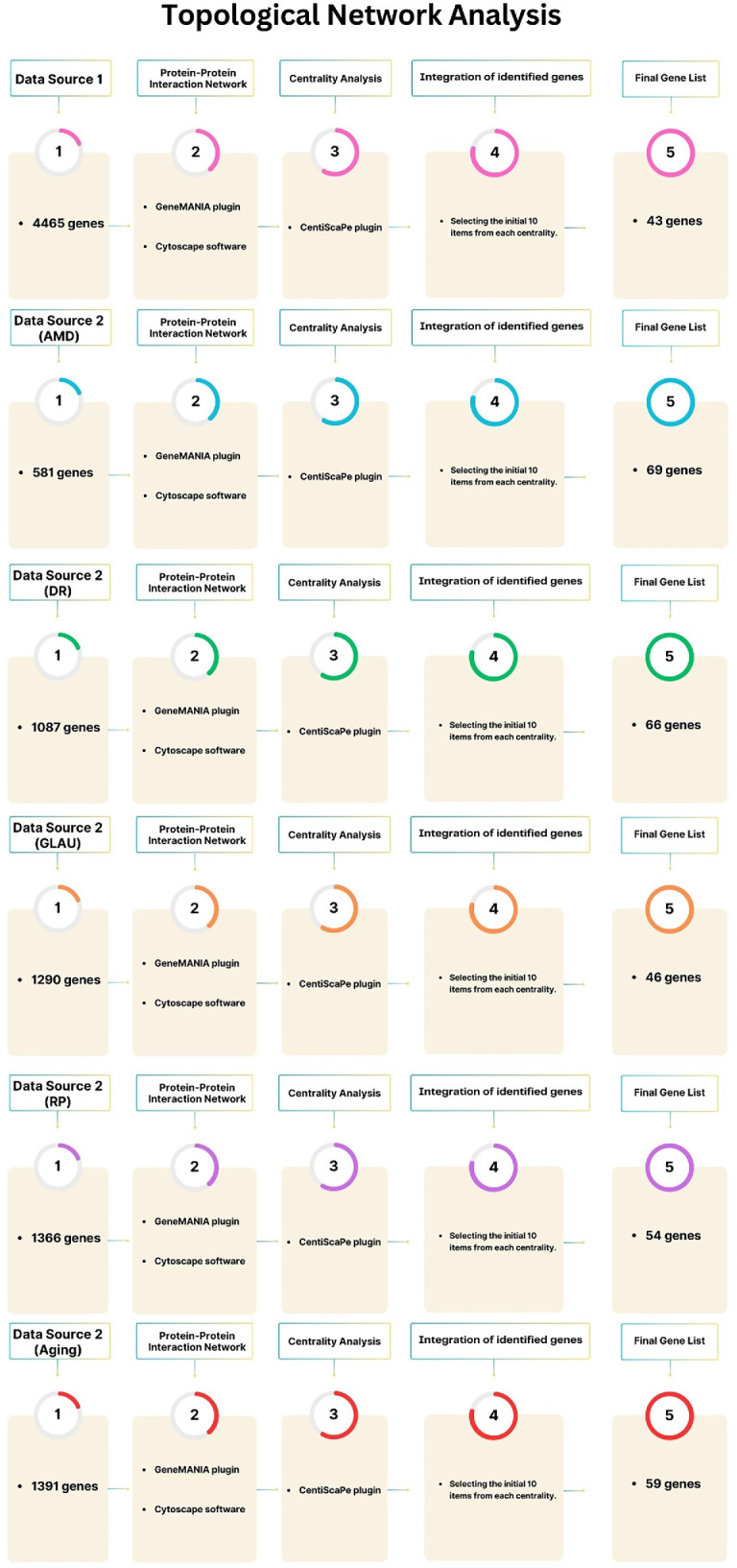
Workflow for topological network analysis and hub gene identification across multiple datasets. It illustrates the sequential pipeline used to analyze gene interaction networks derived from different data sources (Data Source 1 and Data Source 2: AMD, DR, GLAU, RP, and Aging). Each dataset underwent (1) gene collection, (2) construction of protein–protein interaction (PPI) networks using the GeneMANIA plugin and Cytoscape, (3) centrality analysis with CentiScaPe to evaluate network influence, (4) integration of the top 10 genes from each centrality metric, and (5) final prioritization of hub genes. The resulting hub lists ranged from 43 to 69 genes, depending on the dataset, highlighting key regulators potentially involved in disease mechanisms and aging pathways.

To identify key genes from Data Sources 1 and 2 (Supplementary Tables S1–S6), networks were reconstructed using Cytoscape (v3.9.1)^53^ and the GeneMANIA (https://genemania.org/) plugin (v3.5.1).^54^ Hub genes were identified via Network Analyzer and Centiscape 2.2, assessing 12 centrality parameters: degree, betweenness, closeness, centroid, eigenvector, bridging, eccentricity, radiality, stress, topological coefficient, clustering coefficient, and neighborhood connectivity.^55^^–^^58^ This analysis identified 43 key genes from pathway data (Supplementary Table S8), 69 genes for AMD (Supplementary Table S9), 66 for DR (Supplementary Table S10), 46 for GLAU (Supplementary Table S11), 54 genes for RP (Supplementary Table S12), and 59 genes for aging (Supplementary Table S13). The full workflow is illustrated in Figure 4.

#### Genes Identified via Network-Based Drug Prioritization Related to Diseases

To identify proteins targeted by candidate drugs within disease networks of AMD, DR, GLAU, and RP, two ranking algorithms—TrustRank and closeness centrality—were applied via NeDRex-Web (https://web.nedrex.net/)^59^ to protein–protein interaction networks. Drug selection met three criteria: (1) experimentally validated, (2) directly linked to target proteins, and (3) approved by the US Food and Drug Administration (FDA). These algorithms prioritize drugs with a strong impact by targeting multiple key “seed” nodes. Integration of gene–drug relationships revealed 5 key genes for AMD, 7 genes for GLAU, 8 genes for DR, and 26 genes for RP, as shown in Table 2 and Supplementary Table S32.

#### A Two-Tier Network Medicine and Machine Learning Framework Identifies Shared Aging–Angiogenesis Hubs Across Retinal Diseases

**Figure 5.**
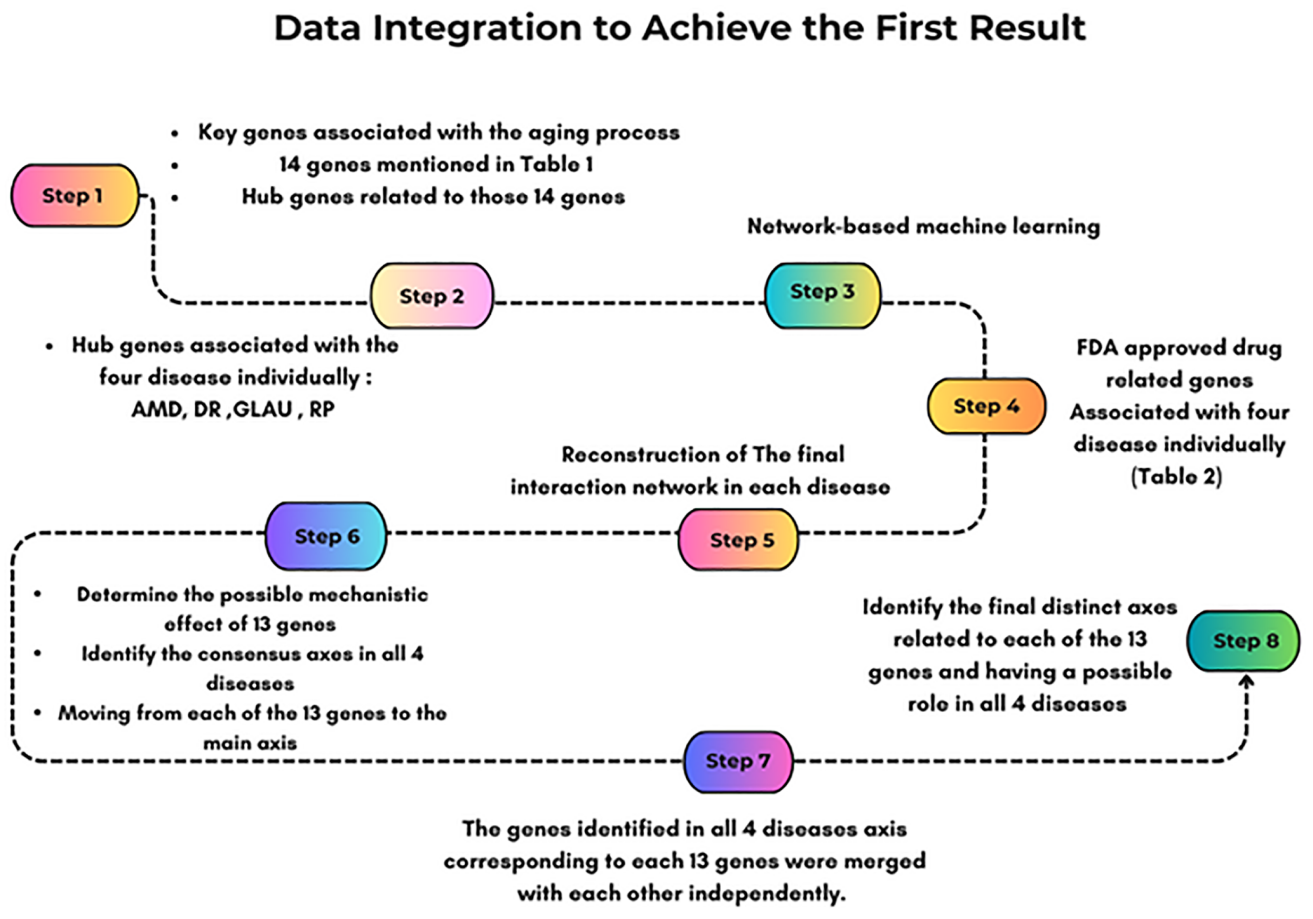
Workflow of the data integration process to identify shared mechanistic axes across aging and ocular diseases. This schematic outlines the eight-step pipeline used to integrate key genes associated with aging and four major eye diseases (AMD, DR, GLAU, RP). Steps included (1) identification of aging-related and hub genes, (2) selection of disease-specific hub genes, (3) application of network-based machine learning, (4) mapping of US Food and Drug Administration–approved drug-related genes, (5) reconstruction of final interaction networks, (6) determination of mechanistic effects and consensus axes across diseases, (7) merging of disease-specific gene axes, and (8) identification of final distinct axes with roles in all four diseases. This integrative approach highlights convergent molecular pathways and therapeutic targets.

Data integration was conducted in eight steps. These included the following: (1) Combined aging-related genes (Supplementary Table S13), 14 pathway-associated genes (Table 1), and their hub genes (Supplementary Table S8). (2) Merged the above with hub genes for AMD, DR, GLAU, and RP (Supplementary Tables S9–S12) independently. (3) Applied network-based machine learning via GenePlexus (https://www.geneplexus.net/)^60^ to each disease list using BioGRID (https://thebiogrid.org/) (v4.2.191),^61^ STRING (v11.0) (https://version-11-0.string-db.org/), and three features (Adjacency, Influence, and Embedding), incorporating DisGeNET (https://disgenet.com/) and Gene Ontology (GO). Input: Supplementary Table S15; Output: disease-specific gene lists (Supplementary Tables S17–S20). Ten novel genes were prioritized based on maximal probability scores of 1. (4) Added FDA-approved drug-related genes (Table 2) to each list to finalize four gene panels (Supplementary Table S33). (5) Reconstructed final disease networks using NetworkAnalyst (https://www.networkanalyst.ca/)^62^ and STRING. (6) Mapped each of the 14 genes to mechanistic axes across all four diseases. (7) Merged genes identified across axes for each gene separately. (8) Reconstructed networks using NetworkAnalyst and IMEx (https://www.imexconsortium.org/) from innateDB (http://www.innatedb.com/)^63^ to define final axes relevant to all four diseases. The full integration flow is shown in Figure 5. The horizontally combined gene sets (Supplementary Fig. S2) were used for pathway enrichment analysis (Supplementary Table S34). We employed a two-tier framework (GenePlexus for network-based gene discovery via a machine learning pipeline and several databases such as NeDRex for network medicine analysis), an innovative combination not previously applied to ocular disease datasets.

**Table 1.** Genes Involved in the Common Pathways Associated With Four Eye Diseases

Gene Name	Description	Gene Name	Description
*ANGPT2*	Angiopoietin 2^121^	*MMP9*	Matrix metallopeptidase 9^122^
*CCL2*	C-C motif chemokine ligand 2^123^	*MMP14*	Matrix metallopeptidase 14^124^
*CXCL1*	C-X-C motif chemokine ligand 1^125^	*STAT3*	Signal transducer and activator of transcription 3^126^
*FOXO1*	Forkhead box O1^127^	*VEGFA*	Vascular endothelial growth factor A^128^
*HSPA5* (*GRP78*)	Heat shock protein family A (Hsp70) member 5^129^	*VEGFC*	Vascular endothelial growth factor C^130^
*IL6*	Interleukin 6^131^	*GRB2*	Growth factor receptor bound protein 2^132^
*MAPK1*	Mitogen-activated protein kinase 1^133^	*DDIT3*	DNA damage inducible transcript 3^134^

**Table 2.** Key Genes Identified Associated With Each Disease Considering the Network-Based Drug Prioritization

Gene Name	AMD	Gene Name	DR	Gene Name	GLAU
*PARP12*	Poly (ADP-ribose) polymerase family member 12	*PON1*	Paraoxonase 1	*MYLK*	Myosin light chain kinase
*VEGFA*	Vascular endothelial growth factor A	*VEGFA*	Vascular endothelial growth factor A	*TEK (TIE2)*	TEK receptor tyrosine kinase
*TLR4*	Toll-like receptor 4	*AGTR1*	Angiotensin II receptor type 1	*PTGS2*	Prostaglandin-endoperoxide synthase 2
*RLBP1*	Retinaldehyde binding protein 1	*CASP3*	Caspase 3	*SLC1A1*	Solute carrier family 1 member 1
*SLC16A8*	Solute carrier family 16 member 8	*ICAM1*	Intercellular adhesion molecule 1	*CYP1B1*	Cytochrome P450 family 1 subfamily B member 1
		*SIRT1*	Sirtuin 1	*CHAT*	Choline O-acetyltransferase
		*GAD2*	Glutamate decarboxylase 2	*CACNA1A*	Calcium voltage-gated channel subunit alpha1 A
		*SOD1*	Superoxide dismutase 1		
**RP**					

**Gene Name**		**Gene Name**		**Gene Name**	

*IMPG2*	Interphotoreceptor matrix proteoglycan 2	*TTPA*	Alpha tocopherol transfer protein	*CA4*	Carbonic anhydrase 4
*PDE6B*	Phosphodiesterase 6B	*AHR*	Aryl hydrocarbon receptor	*IMPDH1*	Inosine monophosphate dehydrogenase 1
*PDE6A*	Phosphodiesterase 6A	*ATF6*	Activating transcription factor 6	*PRPF4*	Pre-mRNA processing factor 4
*CNGA1*	Cyclic nucleotide gated channel subunit alpha 1	*RLBP1*	Retinaldehyde binding protein 1	*RHO (OPN2)*	Rhodopsin
*LRAT*	Lecithin retinol acyltransferase	*IDH3B*	Isocitrate dehydrogenase (NAD (+)) 3 noncatalytic subunit beta	*RBP4*	Retinol binding protein 4
*MERTK*	MER proto-oncogene, tyrosine kinase	*RDH12*	Retinol dehydrogenase 12	*SNRNP200*	Small nuclear ribonucleoprotein U5 subunit 200
*RPE65*	Retinoid isomerohydrolase RPE65	*ABHD12*	Abhydrolase domain containing 12, lysophospholipase	*PDE6G*	Phosphodiesterase 6G
*IDH3A*	Isocitrate dehydrogenase (NAD(+)) 3 catalytic subunit alpha	*CACNA2D4*	Calcium voltage-gated channel auxiliary subunit alpha2delta 4	*RBP3*	Retinol binding protein 3
*NEK2*	NIMA-related kinase 2	*CACNA1F*	Calcium voltage-gated channel subunit alpha1 F		

#### AMD–RPE Single-Cell RNA Sequencing Network Analysis Defines Cluster-Specific Molecular Hubs

Top marker genes for each of the 11 AMD–RPE cell clusters (fibroblast, melanocyte, T/NK cell, Schwann, Schwann 1, RPE, pericytes, endothelial, macrophage, B-cell, mast cell) were independently extracted from the EyeDiseases database (https://eyediseases.bio-data.cn/) (Supplementary Table S7). To identify cluster-specific hub markers relevant to AMD pathogenesis, networks were reconstructed for each cluster using Cytoscape and centrality analysis (12 parameters). The top 10 genes from each parameter were integrated to form 11 hub gene lists (Supplementary Table S14).

Next, the 14 genes from Data Source 1 were combined with the hub genes of each cluster and input into GenePlexus (https://www.geneplexus.net/) (Supplementary Table S16). Using BioGRID (v4.2.191), STRING (v11.0), and three machine learning models (Adjacency, Influence, and Embedding) with DisGeNET and GO, 10 novel genes were selected per cluster based on a probability score of 1 (the maximum value) (Supplementary Tables S21–S31). These were integrated with input genes to form 11 final gene lists representing signaling axes per cluster. Each final list was analyzed in NetworkAnalyst (https://www.networkanalyst.ca/) using IMEx or STRING to trace axes from tested genes to major nodes, including VEGF, yielding 14 axes per cluster (Supplementary Table S36). These vertical axes (Supplementary Fig. S3) were used for pathway enrichment analysis (Supplementary Table S37).

#### False Discovery Rate–Guided Pathway Prioritization Defines Core Shared Axes Across Retinal Diseases

To explore the biological significance of identified genes and uncover shared pathways among the four diseases, pathway enrichment analysis was performed using ExpressAnalyst (https://www.expressanalyst.ca/),^64^ integrating data from KEGG.^65^ A significant cutoff of a false discovery rate (FDR) <0.05 was applied. For four-disease-related enrichment, pathways were analyzed for the presence (1) or absence (0) of experimentally tested genes. Pathways containing 6 to 12 such genes were considered pivotal (Supplementary Table S35). For AMD clusters, the five most significant pathways (based on FDR) were selected across all clusters. These were merged into a comprehensive list, and pathways found in 4 to 10 clusters were considered critical for further analysis (Supplementary Table S38).

### Experimental Studies

#### MiRGD Recombinant Peptide Expression, Purification, and Nanocomplex Assembly for htsFLT01 Gene Delivery

The MiRGD peptide was expressed in *Escherichia* *coli* BL21 carrying the pET28a vector (Novagen).^42^ Cultures were grown in 2xYT medium and induced with 0.5 mM isopropyl β-D-1-thiogalactopyranoside (IPTG). Cells were lysed in denaturing buffer by ice-cooled sonication, then centrifuged. The peptide was purified under denaturing conditions using Ni-NTA affinity chromatography (Qiagen, Hilden, Germany) and analyzed by 15% SDS-PAGE (Tris-glycine). Desalting was carried out via dialysis with a 3.5 kDa MWCO membrane (Sigma-Aldrich, St. Louis, MO, USA).

The MiRGD peptide is a multifunctional cell-penetrating peptide engineered with several distinct motifs: tandem repeats of histone H1 for DNA condensation and packaging, the HIV glycoprotein 41 (Gp41) fusion domain for endosomal escape, the simian virus 40 (SV40) nuclear localization signal for nuclear import, and the 9-amino acid cyclic internalized RGD (iRGD) motif (CRGDKGPDC) for targeted cellular uptake and enhanced tissue penetration.^41^^–^^44^

The iRGD component, a proteolytically activatable variant of the classic RGD peptide, initially binds to αvβ3 and αvβ5 integrins, which are prominently expressed on tumor-associated endothelial cells, various cancer cells, and RPE cells.^66^^–^^68^

Upon binding and subsequent proteolytic cleavage (often by tumor- or tissue-associated proteases), the peptide exposes its C-terminal CendR motif, which then engages neuropilin 1.^67^^,^^68^ This dual-receptor mechanism facilitates deeper tissue penetration and internalization of attached or complexed cargos, such as nanoparticles, drugs, or nucleic acids, while promoting more efficient and selective delivery with potentially reduced off-target effects.

In the context of nonviral gene delivery, MiRGD enables the formation of stable nanocomplexes with plasmid DNA through electrostatic interactions between its positively charged motifs and the negatively charged nucleic acid backbone.

This condensation neutralizes the DNA charge, protects it from nuclease degradation, and yields nanoparticles with optimized physicochemical properties (e.g., size and surface charge) suitable for retinal targeting following intravitreal administration. The htsFLT01 coding sequence was cloned into the pAAV–MCS–IRES–GFP vector^19^ and transformed into *E. coli* XL10. Plasmid extraction followed a maxi prep protocol (Qiagen). MiRGD/htsFLT01 nanocomplexes were then prepared at various nitrogen to phosphate (N/P) ratios using a calculated formula and incubated at room temperature.^41^

#### RPE Cell Culture, Transfection, and Flow Cytometry

An immortalized human RPE cell line was obtained from the NIGEB cell bank (Tehran, Iran). RPE cells were selected to model upstream VEGF resistance mechanisms, rather than downstream endothelial responses. Cells were cultured in Dulbecco's modified Eagle's medium (DMEM)/F12 medium (Gibco, Thermo Fisher Scientific, Waltham, MA, USA) supplemented with 10% fetal bovine serum (FBS; Gibco) under standard conditions (37°C, 5% CO₂, humidified incubator). At passage 8, RPE cells were seeded at 1.5 × 10⁵ cells/well in 24-well plates to reach ∼70% confluency. After a 2-hour incubation in serum-free medium, cells were treated with nanocomplexes at varying N/P ratios in the presence of chloroquine for 4 hours. They were then cultured for 48 hours in DMEM/F12 with 10% FBS. Controls included nontransfected cells and naked htsFLT01 plasmid DNA as the negative control and 25 kDa branched polyethylenimine (PEI) as the positive control. GFP expression was qualitatively assessed using inverted fluorescence microscopy (Zeiss Axiovert, Carl Zeiss, Oberkochen, Germany) and quantified by flow cytometry (BD FACS Calibur; BD Biosciences, San Jose, CA, USA) after trypsinization, PBS wash, and centrifugation (1500 rpm, 5 minutes). A minimum of 10,000 events per sample within the singlet gate were recorded. Untreated cells served as the negative control, and data were analyzed using FlowJo software (Tree Star, Ashland, OR, USA). Results are presented as mean ± SEM from three independent experiments (*n* = 3).

#### Quantitative RT-PCR Analysis

Total RNA from RPE cells incubated with htsFLT01/MiRGD and GFP/MiRGD complexes (used as control) was extracted using TriPure Isolation Reagent (Roche, Mannheim, Germany) according to the manufacturer's instructions at 48 and 72 hours posttransfection. Nontransfected cells were included as the baseline control. Following treatment with DNaseΙ enzyme (ABMgood, Richmond, BC, Canada), reverse transcription and amplification were performed using the Revert Aid First Strand cDNA Synthesis Kit (Thermo Fisher Scientific, Waltham, MA, USA). Quantitative RT-PCR (RT-qPCR) was performed by the QuantiFast SYBR Green PCR Kit (Qiagen) with a Real-Time PCR system (Applied Biosystems, Foster City, CA, USA) to quantify the expression levels of the target genes. The specific primer sequences are listed in Table 3. The method of performing the test began with PCR initial heat activation at 95°C for 5 minutes, followed by two-step cycling, denaturation at 95°C, and combined annealing/extension at 60°C, with 15 and 40 seconds for 45 cycles, respectively. The internal control gene GAPDH was used to normalize the expression levels of the candidate genes. Next, the relative expression levels of the genes were calculated using the 2^–^^ΔΔCT^ method. The experimental design considered both technical and biological replicates, which involved three independent duplicates.

**Table 3.** The Primer Sequences of the Studied Genes

Gene Name	Forward Primer	Reverse Primer
*GAPDH*	GCACCACCAACTGCTTAGC	GGCATGGACTGTGGTCATGA
*MMP14*	GAGCTCAGGGCAGTGGATAG	GGTAGCCCGGTTCTACCTTC
*IL6*	ACTCACCTCTTCAGAACGAATTG	CCATCTTTGGAAGGTTCAGGTTG
*STAT3*	ATCACGCCTTCTACAGACTGC	CATCCTGGAGATTCTCTACCACT
*FOXO1*	TCGTCATAATCTGTCCCTACACA	CGGCTTCGGCTCTTAGCAAA
*ANGPT2*	ACCCCACTGTTGCTAAAGAAGA	CCATCCTCACGTCGCTGAATA
*MMP9*	CTTTGACAGCGACAAGAAGTGG	ATGCCATTCACGTCGTCCTTAT
*VEGFC*	AGTTCCACCACCAAACATGC	TGAAGGGACACAACGACACA
*HSPA5* (*GRP78*)	CGTGGAGATCATCGCCAAC	ACATAGGACGGCGTGATGC
*CCL2*	CAGCCAGATGCAATCAATGCC	TGGAATCCTGAACCCACTTCT
*GRB2*	ATTCCTGCGGGACATAGAACA	GGTGACATAATTGCGGGGAAAC
*VEGFA*	AGGGCAGAATCATCACGAAGT	AGGGTCTCGATTGGATGGCA
*CXCL1*	AAGTCATAGCCACACTCAAG	TTGTCACTGTTCAGCATCTT
*MAPK1*	TACACCAACCTCTCGTACATCG	CATGTCTGAAGCGCAGTAAGATT

#### Statistical Analysis

The data were presented as mean ± SEM, derived from three independent tests. RT-qPCR analysis was conducted using three independent duplicate samples. Student's *t*-test was used to compare differences between the experimental and control groups. A *P* value of less than 0.05 was considered statistically significant. GraphPad Prism version 10.2.3 (GraphPad Software) was used for all statistical analysis and graphical representations.

## Results

### Final Axes Linking 14 Key Genes Across All Four Eye Diseases

**Figure 6.**
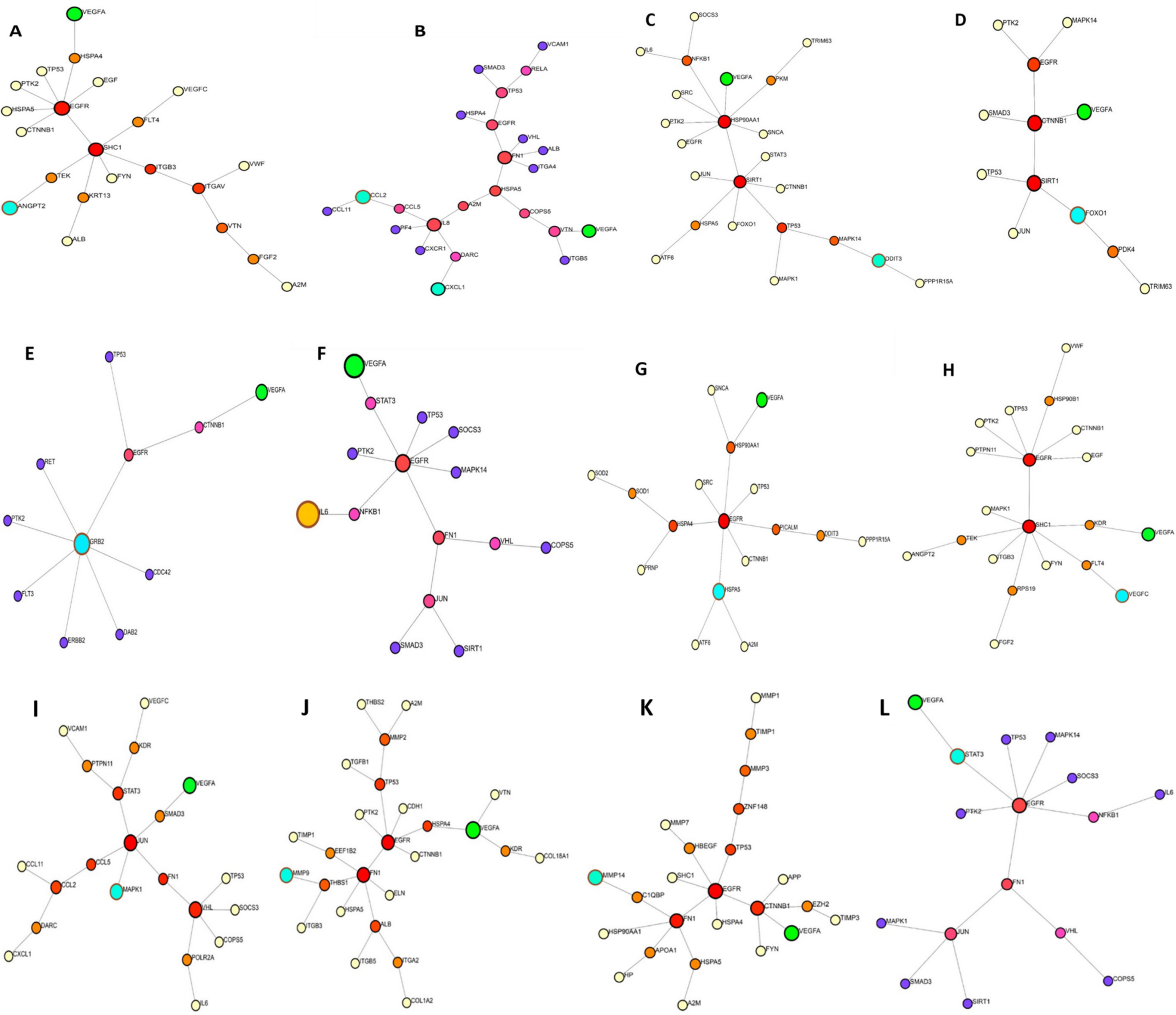
Key molecular axes linking VEGF to htsFLT01 targets validated by qPCR. Identification of critical signaling axes connecting VEGF, the primary angiogenic driver and target of htsFLT01, to downstream genes implicated in ocular disease pathology. Data integration highlighted disease-relevant targets, which were subsequently evaluated by qPCR. Panels show individual axes: (**A**) VEGF–ANGPT2, (**B**) VEGF–CCL2/CXCL1, (**C**) VEGF–DDIT3, (**D**) VEGF–FOXO1, (**E**) VEGF–GRB2, (**F**) VEGF–IL6, (**G**) VEGF–HSPA5, (**H**) VEGF–VEGFC, (**I**) VEGF–MAPK1, (**J**) VEGF–MMP9, (**K**) VEGF–MMP14, and (**L**) VEGF–STAT3. Together, these axes define key mechanistic connections between VEGF signaling and gene networks driving pathological angiogenesis and inflammation.

The distinct interaction axes for each of the 14 experimentally tested genes implicated in AMD, DR, GLAU, and RP are visualized across the figures, with a comprehensive overview provided in Figure 6.

**Figure 7.**
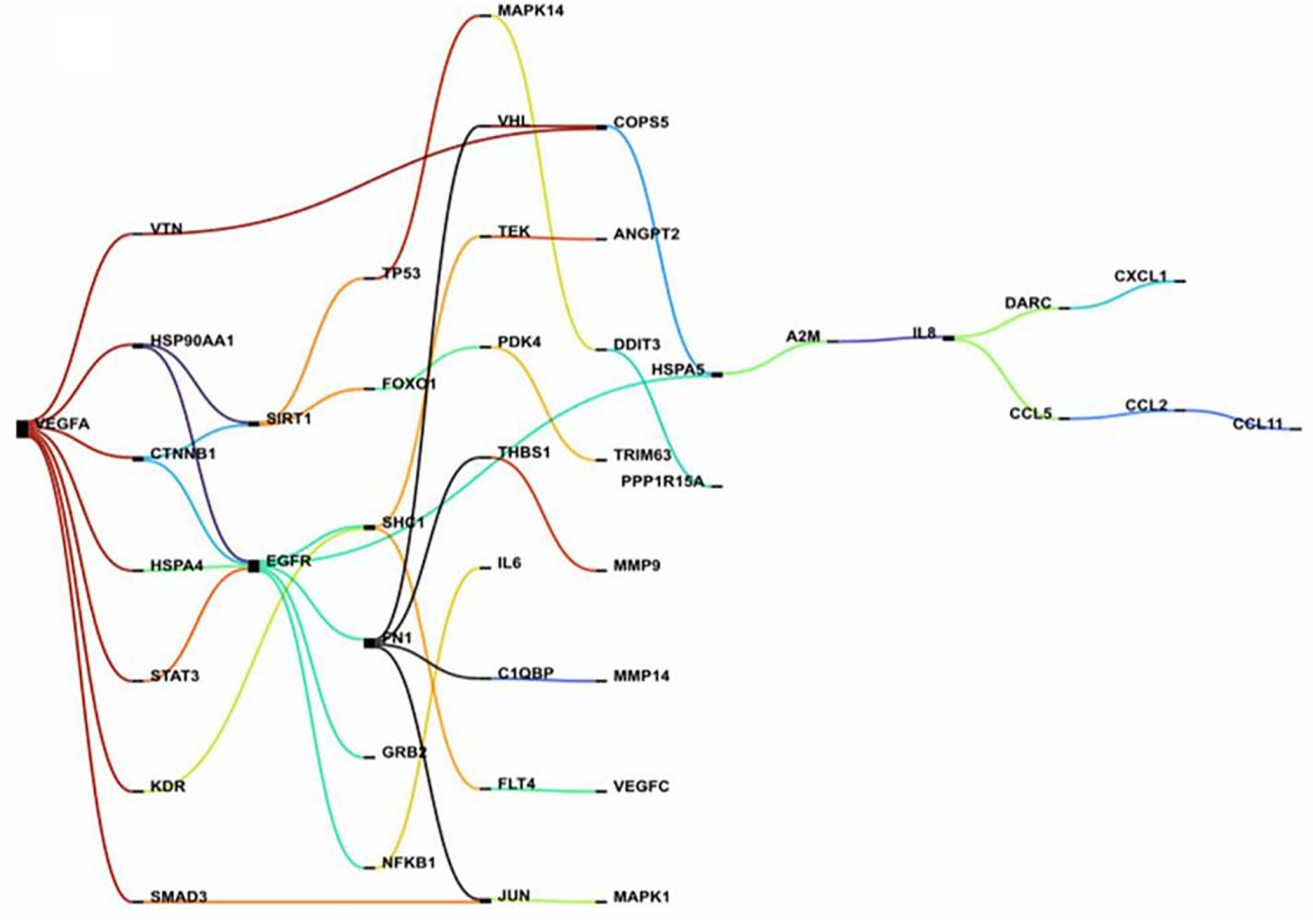
Visualization of VEGF–gene interaction networks through the Sankey diagram. The Sankey diagram demonstrates the directional flow of interactions from VEGFA toward downstream targets, highlighting gene connectivity and relative weight within signaling axes. The diagram provides complementary insights into how VEGF signaling is distributed across multiple target pathways, emphasizing potential regulatory hubs relevant to pathological angiogenesis and therapeutic intervention.

The Sankey diagram in Figure 7 reveals that VEGF inhibition exerts antiangiogenic effects through its seven closest neighbors: VTN, HSP90AA1, CTNNB1, KDR, HSPA4, STAT3, and SMAD3. In parallel, EGFR and SIRT1 emerge as key players, closely interacting with the VEGF–VEGFR2 axis and contributing to inflammation, MMP activation, and lack of response to anti-VEGF. To better illustrate the interconnections, a chord diagram in Supplementary Figure S5 maps the interactions among multiple nodes. Together, both the Sankey and chord diagrams offer a complementary perspective on how VEGF-targeted therapies interface with the broader disease molecular mechanisms.

### Classification of Shared Pathways Among Four Eye Diseases

Analysis of pathway enrichment data across AMD, DR, GLAU, and RP revealed 21 pivotal pathways, which were grouped into six major categories (Table 4). (1) Inflammation and Immune Response: TNF, IL-17, Toll-like receptor, and chemokine signaling. (2) Vascular Function and Remodeling: VEGF signaling, better or suboptimal responders to EGFR inhibitors, and fluid shear stress. (3) Cell Growth and Survival: PI3K–Akt, MAPK, HIF-1, and Ras signaling. (4) Cell Communication and Adhesion: Adherens junctions, focal adhesion, actin cytoskeleton regulation, and Rap1 signaling. (5) Metabolism and Cellular Stress Response: Cellular senescence, FoxO, and AGE–RAGE pathways. (6) Developmental Signaling: Stem cell pluripotency, neurotrophin, and ErbB signaling. These clustered pathways highlight the shared disease mechanisms and therapeutic targets across the four conditions. To further illustrate their relationships with genes assessed by quantitative PCR, a chord diagram was constructed (Supplementary Fig. S6).

**Table 4.** Verifying the Presence (1) or Absence (0) of Genes Assessed by qPCR Within the Four Disease-Related Pathway Enrichment Analysis

Enrichment/Genes	*ANGPT2*	*CXCL1/CCL2*	*DDIT3*	*FOXO1*	*GRB2*	*HSPA5*	*IL6*	*MAPK1*	*MMP9*	*MMP14*	*STAT3*	*VEGFC*	Final
Fluid shear stress and atherosclerosis	1	1	1	1	1	1	1	1	1	1	1	1	12
Focal adhesion	1	1	1	1	1	1	0	1	1	1	1	1	11
PI3K-Akt signaling pathway	1	1	1	1	1	0	1	1	1	1	1	1	11
HIF-1 signaling pathway	1	1	1	1	1	0	1	1	1	1	1	1	11
Rap1 signaling pathway	1	0	1	1	1	1	1	1	1	0	1	1	10
EGFR tyrosine kinase inhibitor resistance	1	0	1	1	1	1	1	1	0	1	1	1	10
Adherens junction	1	0	1	1	1	1	1	0	1	1	1	1	10
MAPK signaling pathway	1	0	1	1	1	0	1	1	0	0	1	1	8
AGE-RAGE signaling pathway in diabetic complications	0	1	1	1	0	0	1	1	1	0	1	1	8
Ras signaling pathway	1	0	1	0	1	0	1	1	0	0	1	1	7
Regulation of actin cytoskeleton	1	0	1	0	1	0	1	0	1	0	1	1	7
ErbB signaling pathway	1	0	1	1	1	0	1	0	0	0	1	1	7
IL-17 signaling pathway	0	1	1	1	0	0	1	1	0	1	1	0	7
VEGF signaling pathway	0	0	1	1	1	0	1	1	0	0	1	1	7
Neurotrophin signaling pathway	0	0	1	1	1	0	1	1	0	0	1	1	7
Chemokine signaling pathway	0	1	1	0	1	0	1	1	0	0	1	0	6
TNF signaling pathway	0	1	1	1	0	0	1	1	0	0	1	0	6
Cellular senescence	0	1	1	1	0	0	1	1	0	0	1	0	6
FoxO signaling pathway	0	0	1	1	0	0	1	1	0	0	1	1	6
Toll-like receptor signaling pathway	0	0	1	1	1	0	1	1	0	0	1	0	6
Signaling pathways regulating pluripotency of stem cells	0	0	1	1	0	0	1	1	0	0	1	1	6

### Identification of Key Axis Related to Each of the 14 Genes in RPE Cell Cluster

**Figure 8.**
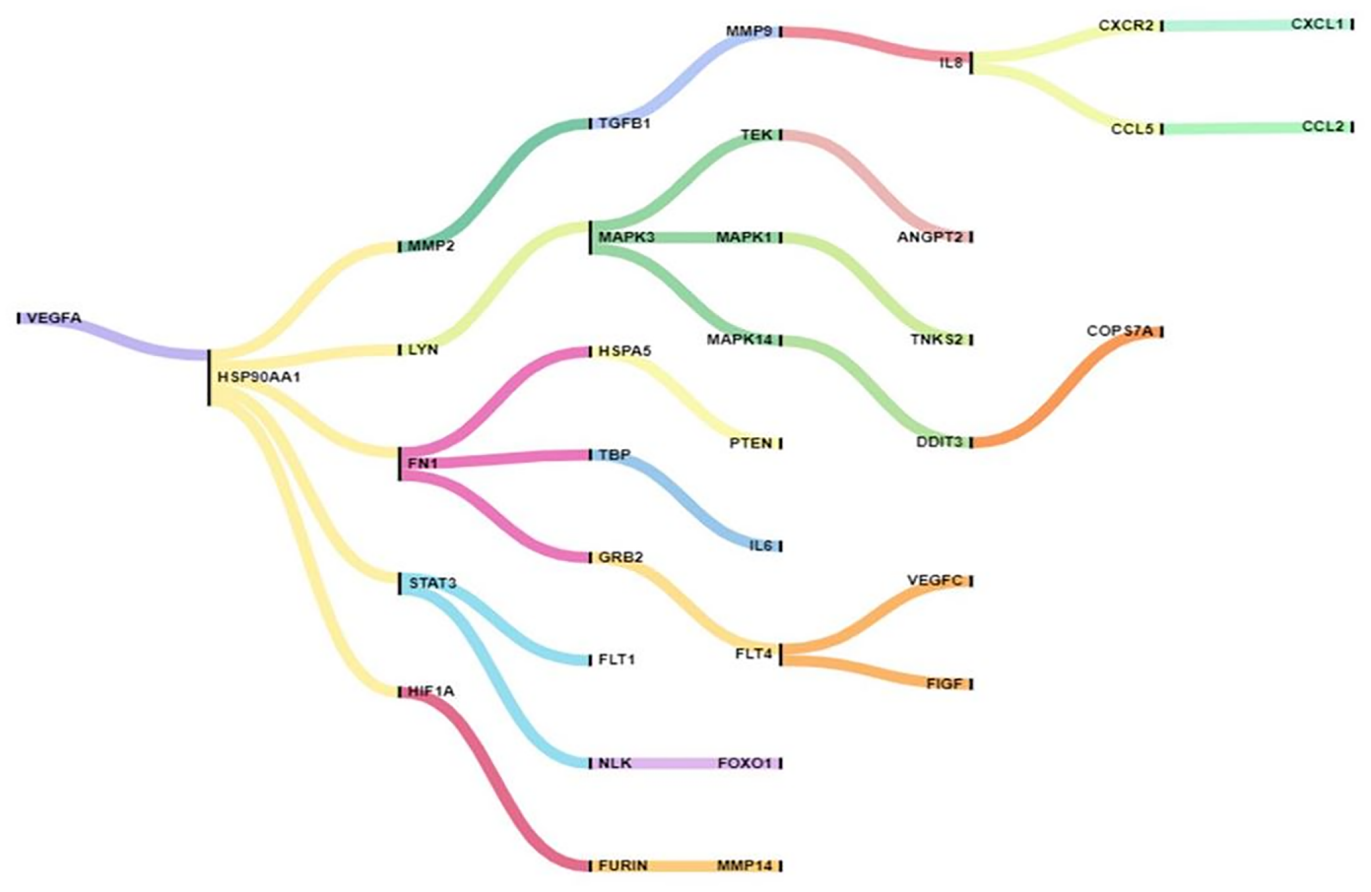
Sankey diagram of VEGFA-centered gene interactions in the RPE cluster. VEGFA serves as the primary node, branching to central hubs such as HSP90AA1, STAT3, and HIF1A, which further connect to downstream genes, including MAPK3, FN1, IL8, FLT4, MMP9, and others. Colored flows represent interaction pathways, with branching indicating cross-regulatory links that drive angiogenic, inflammatory, and signaling processes in the RPE microenvironment.

The analysis of the generated Sankey and Chord diagram, focusing on 14 genes within the RPE cell cluster, indicated that neutralizing VEGF with anti-VEGF molecules plays a crucial role in preventing angiogenesis through influencing HSP90AA1 and its five nearest neighbors, including MMP2, LYN, FN1, STAT3, and HIF1A (Fig. 8; Supplementary Fig. S7). Additionally, a set of potential axes were identified that mediate the intricate relationship between angiogenesis and inflammation. Furthermore, communication bridges related to compensatory pathways were recognized, which could facilitate the emergence of lack of response to anti-VEGF agents. These findings enhanced our mechanistic understanding of how RPE cells respond to anti-VEGF treatments.

### Pathway Enrichment Analysis Related to AMD–RPE Clusters

The results of the enrichment analysis concerning 14 genes and clusters involved in AMD were evaluated based on two steps. First, we identified exclusive signaling pathways related to the RPE cluster, notably including the AGE–RAGE signaling pathway, the TNF signaling pathway, the HIF-1 signaling pathway, the chemokine signaling pathway, and the FoxO signaling pathway. In the next step, we analyzed the enrichment results across all clusters simultaneously. The work aimed to pinpoint the signaling pathways that appeared most frequently across all clusters. These findings indicate that seven signaling pathways, including the AGE–RAGE signaling pathway, TNF signaling pathway, PI3K–Akt signaling pathway, EGFR tyrosine kinase inhibitor better or suboptimal responders, HIF-1 signaling pathway, focal adhesion, and MAPK signaling pathway, play a pivotal role in all clusters (Table 5). It is evident that the pathways with the highest frequency across all clusters associated with AMD disease significantly contribute alongside the exclusive pathways specific to RPE clusters.

**Table 5.** Verifying the Presence (1) or Absence (0) of Genes Assessed by qPCR Within the AMD-RPE Cluster-Related Pathway Enrichment Analysis

Enrichment/Cluster	RPE Cell	Macrophage	Pericyte	Endothelial	B Cell	Mast Cell	Melanocyte	T/NK Cell	Schwann	Schwann 1	Fibroblast	Final
AGE-RAGE signaling pathway in diabetic complications	1	1	1	1	1	1	1	0	1	1	1	10
TNF signaling pathway	1	1	1	0	1	1	1	1	1	1	1	10
PI3K-Akt signaling pathway	0	1	1	0	1	0	1	0	0	1	1	6
EGFR tyrosine kinase inhibitor resistance	0	0	1	1	0	0	1	1	1	1	0	6
HIF-1 signaling pathway	1	0	0	0	0	0	1	1	1	0	1	5
Focal adhesion	0	1	1	1	0	0	0	0	0	1	1	5
MAPK signaling pathway	0	0	0	0	1	1	0	1	1	0	0	4

### Summary of Results

To gain a clear understanding of the outcomes related to the bioinformatics phase, all results were compiled into two tables. Table 6 presents four disease-related consensus axes and RPE cluster-related axes corresponding to 14 experimentally investigated genes. Table 7 outlines the consensus clusters and pathways associated with the four diseases, as well as pathways related to the RPE cluster and those with the highest frequency across all clusters.

**Table 6.** Consensus Axes Related to Disease and RPE Cluster Corresponding to the 14 Experimentally Investigated Genes

Gene	Four Disease-Related Consensus Axes	RPE Cluster-Related Axes
*ANGPT2*	VEGFA, HSPA4, EGFR, SHC1, TEK, ANGPT2	VEGFA, HSP90AA1, LYN, MAPK3, TEK, ANGPT2
*CCL2*	VEGFA, VTN, COPS5, HSPA5, A2M, IL8, CCL5, CCL2, CCL11	VEGFA, HSP90AA1, MMP2, TGFB1, MMP9, IL8, CCL5, CCL2
*MAPK1*	VEGFA, SMAD3, JUN, MAPK1	VEGFA, HSP90AA1, LYN, MAPK3, MAPK1, TNKS2
*FOXO1*	VEGFA, CTNNB1, SIRT1, FOXO1, PDK4, TRIM63	VEGFA, HSP90AA1, STAT3
		-NLK, FOXO1
		-FLT1
*IL6*	VEGFA, STAT3, EGFR, NFKB1, IL6	VEGFA, HSP90AA1, FN1, TBP, IL6
*MMP14*	VEGFA, CTNNB1, EGFR, FN1, C1QBP, MMP14	VEGFA, HSP90AA1, HIF1A, FURIN, MMP14
*CXCL1*	VEGFA, VTN, COPS5, HSPA5, A2M, IL8, DARC, CXCL1	VEGFA, HSP90AA1, MMP2, TGFB1, MMP9, IL8, CXCR2, CXCL1
*HSPA5 (GRP78)*	VEGFA, HSP90AA1, EGFR, HSPA5	VEGFA, HSP90AA1, FN1, HSPA5, PTEN
*MMP9*	VEGFA, HSPA4, EGFR, FN1, THBS1, MMP9	VEGFA, HSP90AA1, MMP2, TGFB1, MMP9, IL8
		-CCL5, CCL2
		-CXCR2, CXCL1
*VEGFA*	—	VEGFA, HSP90AA1, FN1
*STAT3*	VEGFA, STAT3, EGFR	VEGFA, HSP90AA1, STAT3
		-FLT1
		-NLK, FOXO1
*VEGFC*	VEGFA, KDR, SHC1, FLT4, VEGFC	VEGFA, HSP90AA1, FN1, GRB2, FLT4
		-VEGFC
		-FIGF
*GRB2*	VEGFA, CTNNB1, EGFR, GRB2	VEGFA, HSP90AA1, FN1, GRB2, FLT4
		-VEGFC
		-FIGF
*DDIT3*	VEGFA, HSP90AA1, SIRT1, TP53, MAPK14, DDIT3, PPP1R15A	VEGFA, HSP90AA1, LYN, MAPK3, MAPK14, DDIT3, COPS7A

**Table 7.** Consensus Clusters and Pathways Linked to the Four Diseases, RPE Cluster, and Those Occurring Most Frequently Across All Clusters

Consensus Pathway Related to Four Diseases			
Clusters	Pathways	Pathways Related to RPE Cluster	Pathways With Highest Frequency in All Clusters
Inflammation and immune response	TNF signaling pathwayIL-17 signaling pathwayToll-like receptor signaling pathwayChemokine signaling pathway	AGE-RAGE signaling pathway in diabetes complicationsHIF-1 signaling pathwayTNF signaling pathwayFoxO signaling pathwayChemokine signaling pathway	AGE-RAGE signaling pathway in diabetes complicationsHIF-1 signaling pathwayTNF signaling pathwayPI3K-Akt signaling pathwayEGFR tyrosine kinase inhibitor resistanceFocal adhesionMAPK signaling pathway
Vascular function and remodeling	VEGF signaling pathway		
	EGFR tyrosine kinase inhibitor resistance		
	Fluid shear stress		
Cell growth and survival	PI3K-Akt signaling pathway		
	MAPK signaling pathway		
	HIF-1 signaling pathway		
	Ras signaling pathway		
Cell communication and adhesion	Adherens junction		
	Focal adhesion		
	Regulation of actin cytoskeleton		
	Rap1 signaling pathway		
Metabolism and cellular stress response	Cellular Senescence		
	FoxO signaling pathway		
	AGE-RAGE signaling pathway		
Developmental signaling	Signaling pathways regulating pluripotency of stem cells		
	Neurotrophin signaling pathway		
	ErbB signaling pathway		

### Time-Dependent Gene Delivery Efficiency Using MiRGD Peptide in RPE Cells

**Figure 9.**
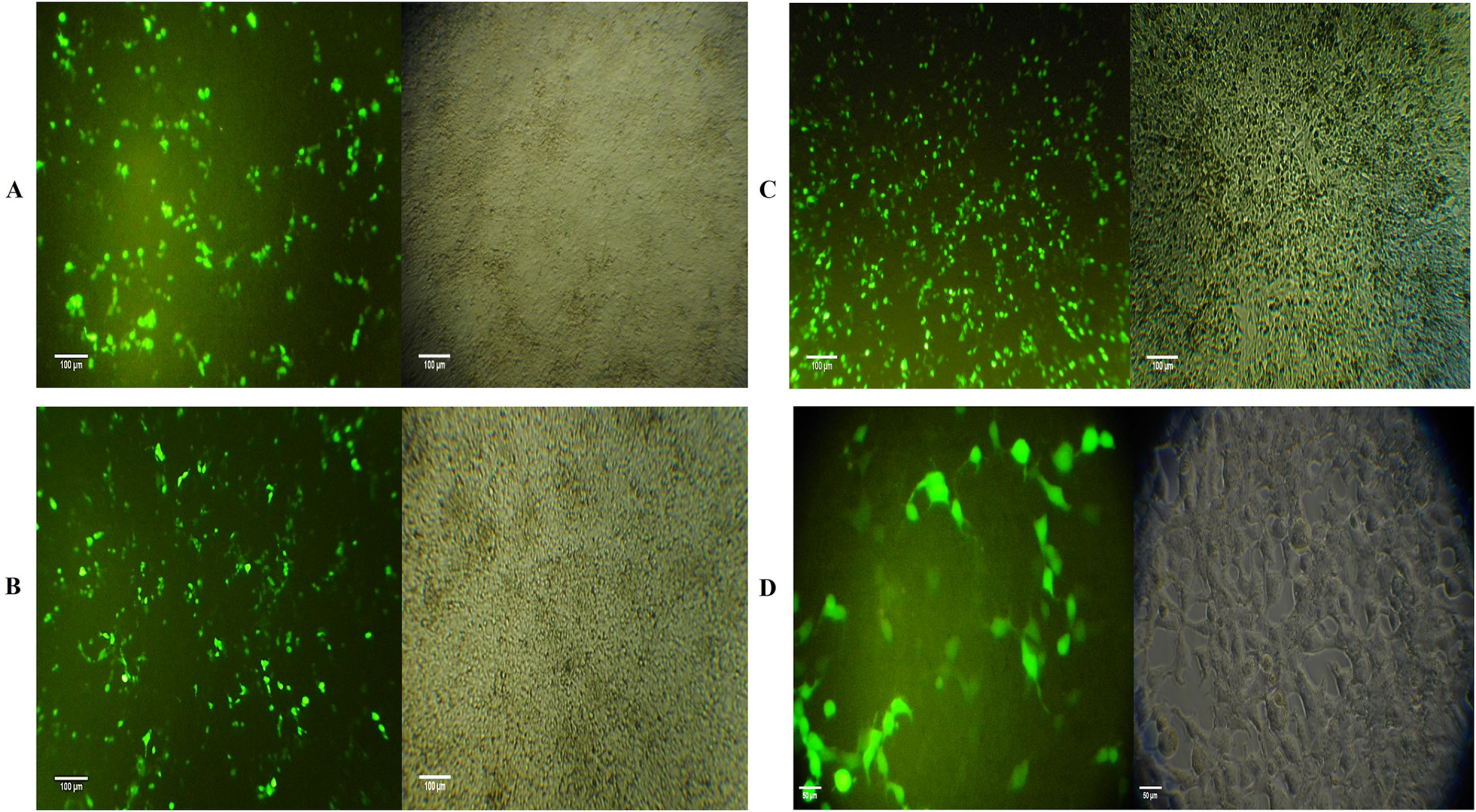
Transfection efficiency of the MiRGD/htsFLT01 complex in human RPE cells. Human RPE cells were transfected with the MiRGD/htsFLT01 complex (N/P 16) and examined after (**A**) 48 hours and (**B**) 72 hours. (**C**) The PEI/htsFLT01 complex was used as a positive control. Images were acquired using an inverted fluorescence microscope (*scale bar*: 100 µm). (**D**) Higher-magnification visualization (200×) of RPE cells transfected with the MiRGD/htsFLT01 complex (N/P 16) highlights intracellular localization (*scale bar*: 50 µm).

The MiRGD fusion peptide had a molecular weight of 9.6 kDa, confirmed by a distinct band on SDS-PAGE (Supplementary Fig. S4). Its ability to deliver the htsFLT01 gene into human RPE (hRPE) cells was tested using various N/P ratios. The highest efficiency was seen at an N/P ratio of 16 with 100 µM chloroquine after 48 and 72 hours (Figs. 9A, 9B). The PEI/htsFLT01 complex served as a positive control (Fig. 9C) and the naked plasmid as a negative control. High-resolution imaging showed successful delivery at 200× magnification (Fig. 9D).

**Figure 10.**
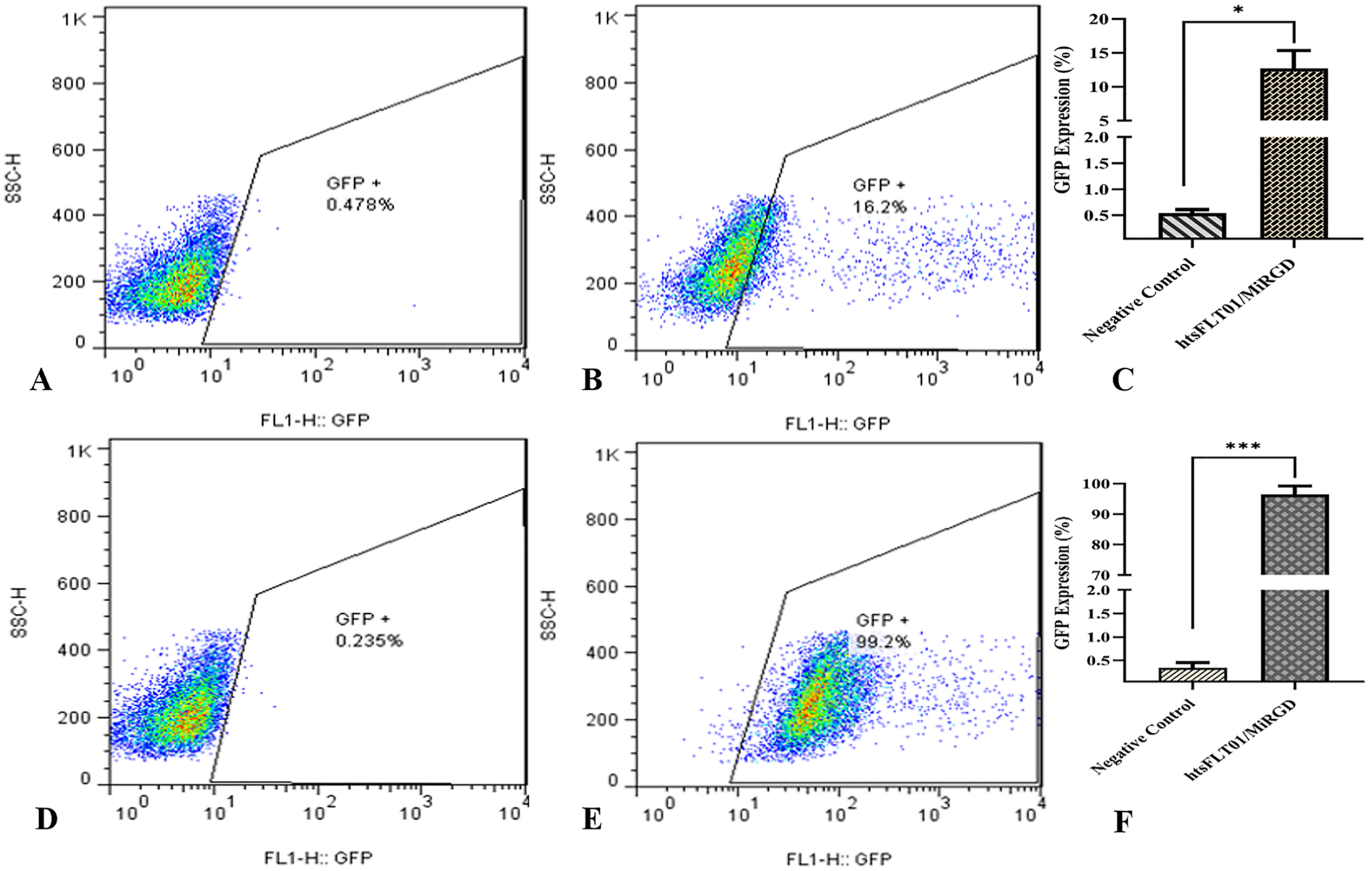
Comparative analysis of GFP expression in RPE cells following htsFLT01/MiRGD transfection. GFP expression was assessed in (**A**) untreated cells (negative control) and (**B**) RPE cells transfected with htsFLT01/MiRGD (N/P 16) after 48 hours, with (**C**) quantification of average GFP-positive cells. At 72 hours posttransfection, GFP expression was evaluated in (**D**) untreated cells (negative control) and (**E**) htsFLT01/MiRGD (N/P 16)–treated cells, with (**F**) quantification of average GFP-positive cells. Data are presented as mean ± SD (*n* = 3) and were analyzed using an unpaired *t*-test (*P < 0.05, ***P < 0.0001).

Flow cytometry quantified GFP expression over time. At 48 hours, the MiRGD/htsFLT01 complex (N/P 16) resulted in 13% GFP-positive cells, significantly above baseline (*P* < 0.05, Figs. 10A–C). At 72 hours, GFP expression increased to 96%, again statistically significant (*P* < 0.05, Figs. 10D–F), confirming enhanced transgene delivery over time.

### Temporal Expression of Candidate Genes After htsFLT01/MiRGD Transfection

**Figure 11.**
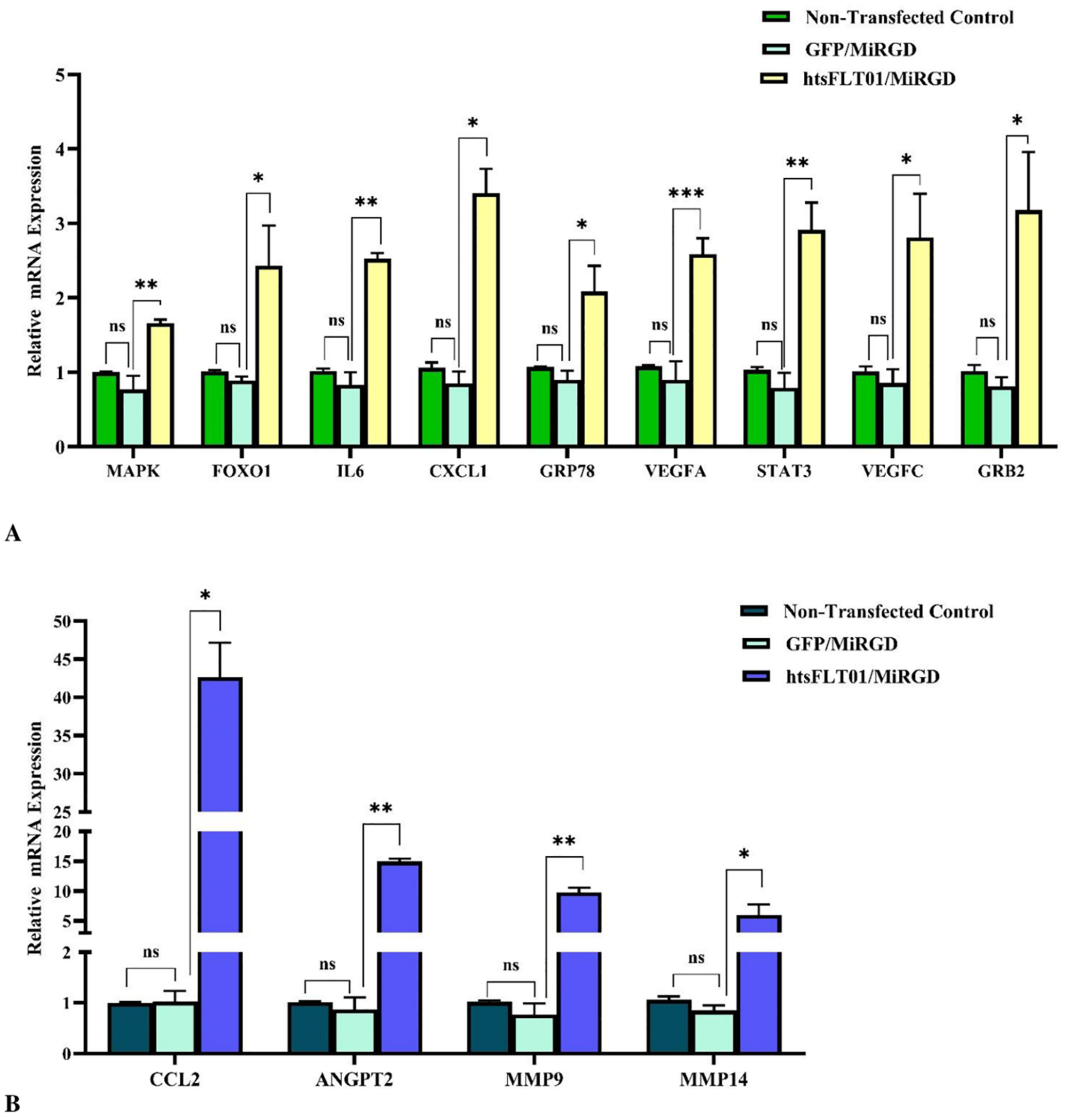
Relative mRNA expression of candidate genes in human RPE cells following incubation with the htsFLT01/MiRGD complex after 48 hours (**A**, **B**). Expression levels are normalized to the reference gene and presented relative to the untreated control. Nontransfected cells were included as a baseline control. No significant differences (*P* < 0.05) were detected between nontransfected cells and mock-transfected cells (GFP/MiRGD). Data represent the mean ± SEM (*n* = 3). Statistical significance was assessed by unpaired two-tailed Student's *t*-test (*P < 0.05, **P < 0.001, ***P < 0.0001; ns, not significant).

**Figure 12.**
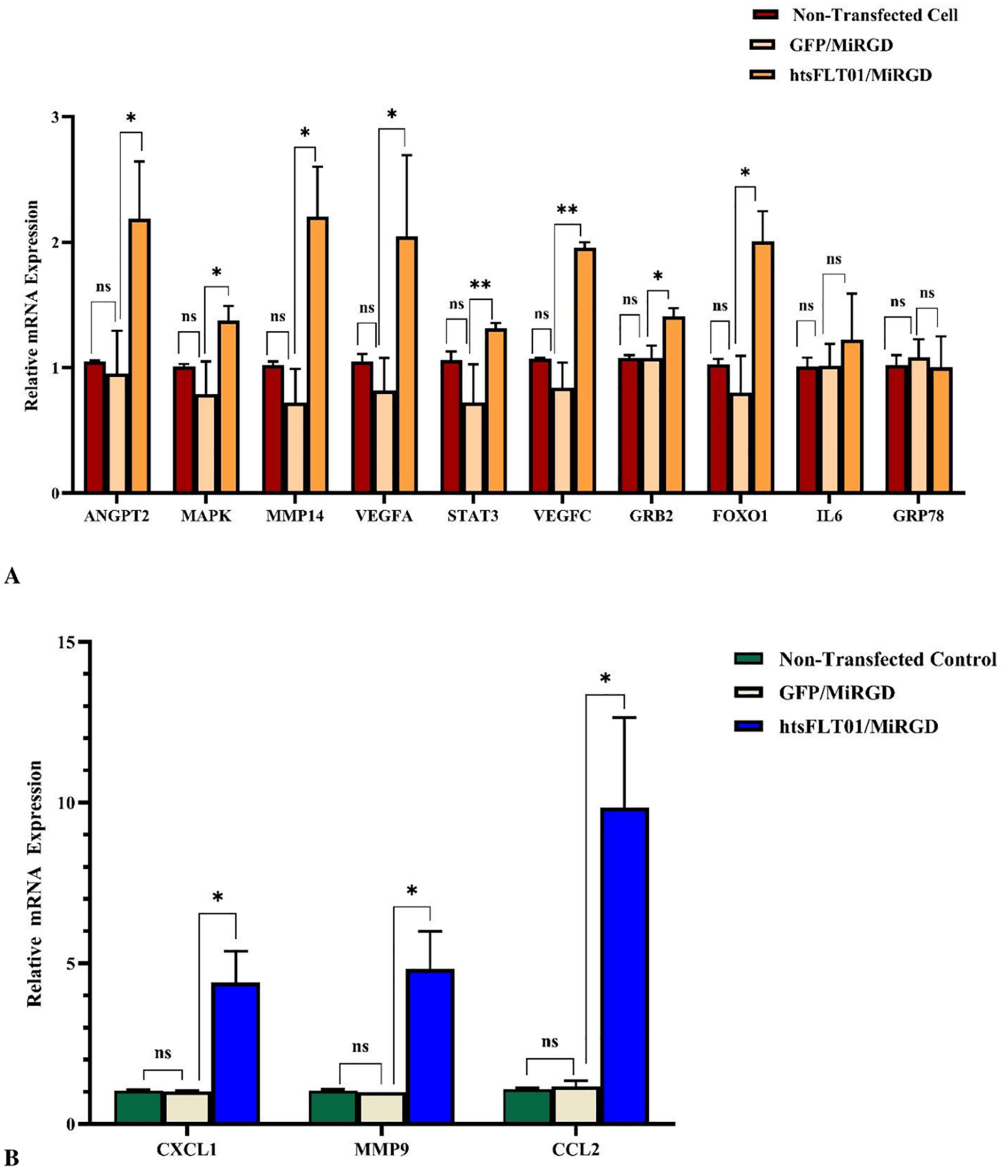
Relative mRNA expression levels of candidate genes were evaluated in human RPE cells at 72 hours posttransfection with the htsFLT01/MiRGD complex (**A**, **B**). mRNA levels were normalized to the appropriate reference gene and expressed relative to untreated (nontransfected) cells as a baseline control. Expression levels did not differ significantly between nontransfected cells and GFP/MiRGD (mock-transfected) controls. Data are presented as mean ± SEM (*n* = 3). Statistical comparisons were performed using unpaired two-tailed Student's *t*-test (*P < 0.05, **P < 0.001; ns, not significant).

**Figure 13.**
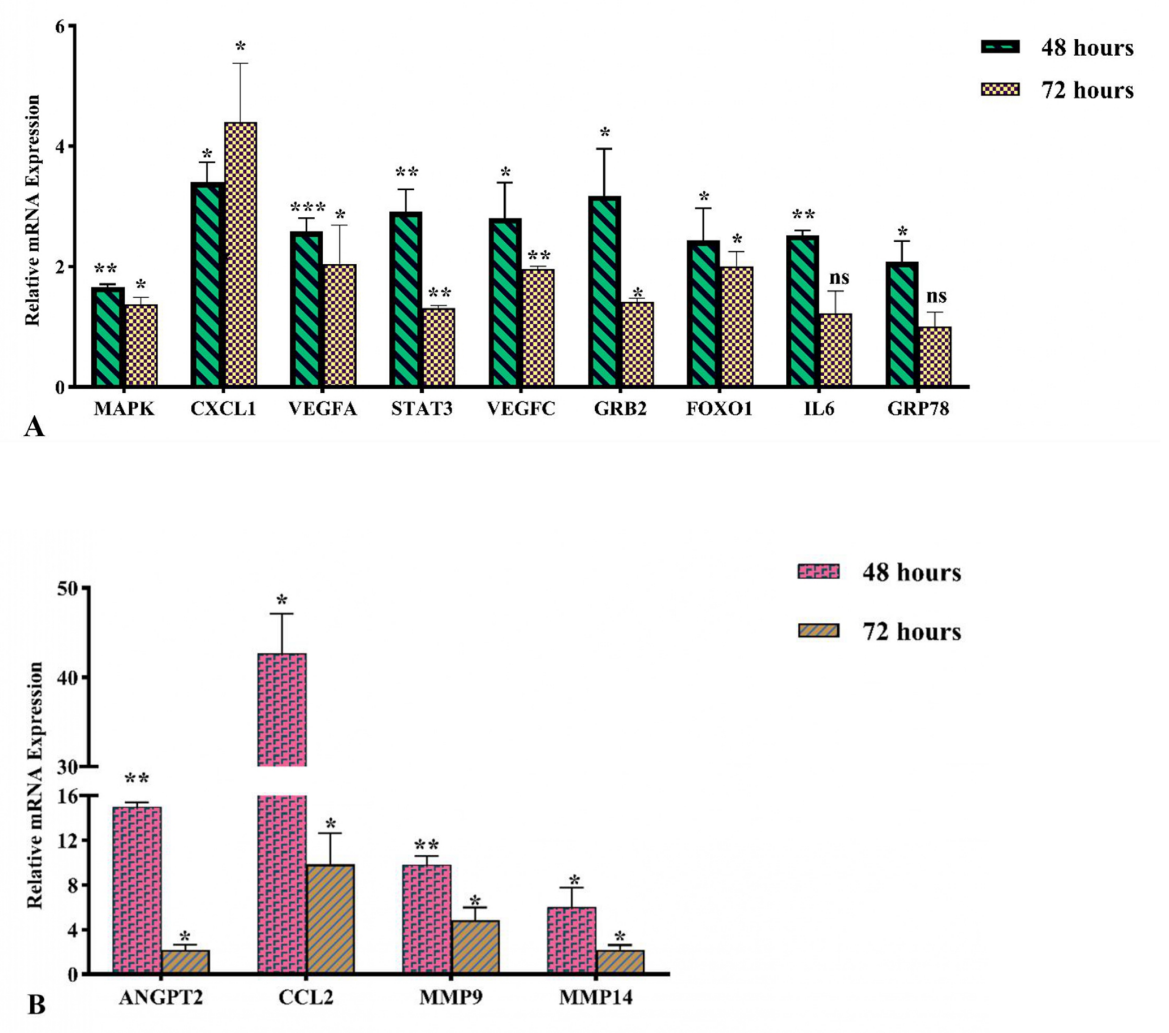
Relative mRNA expression of candidate genes in human RPE cells incubated with the htsFLT01/MiRGD complex was evaluated at two different time points: 48 and 72 hours (**A**, **B**). The data are presented as the mean ± SEM (*n* = 3) and were statistically analyzed using an unpaired *t*-test (*P < 0.05, **P < 0.001, ***P < 0.0001; ns, not significant).

All experiments were conducted under basal culture conditions; therefore, observed effects reflect intrinsic regulatory responses rather than disease-induced states. To evaluate downstream effects of the htsFLT01/MiRGD nanocomplex, quantitative PCR (qPCR) analysis was performed on RPE cells at 48 and 72 hours posttransfection. Candidate genes were selected from the literature (Supplementary Table S39). The gene expression level revealed no significant differences between nontransfected and mock-transfected (GFP/MiRGD) cells across all tested genes at two different time points (Figs. 11, 12). So, the gene expression levels were compared between cells transfected with the htsFLT01-containing construct/MiRGD carrier and those transfected with the corresponding empty vector (GFP/MiRGD-transfected cells used as the control). Compared to GFP/MiRGD-treated control cells, most genes showed significantly elevated expression at both time points (*P* < 0.05–0.001) (Fig. 13; Supplementary Table S39). However, by 72 hours, expression levels generally declined compared to 48 hours, except for *CXCL1*, which continued to rise. No significant changes were detected for *IL6* and *GRP78* at 72 hours (Fig. 13A). Shapiro–Wilk test confirmed normal distribution; the control fold change was set to 1, and differences were analyzed using the unpaired *t*-test. Data are shown as mean ± SEM, with significance noted as *P < 0.05, **P < 0.01, and ***P < 0.001.

## Discussion

This study integrates systems biology, machine learning, and experimental validation to delineate molecular axes associated with suboptimal anti-VEGF responsiveness, as well as cross-disease hypotheses, across multiple retinal diseases, including nAMD, DR, GLAU, and RP. While our systems analysis encompassed glaucoma and retinitis pigmentosa to explore shared aging- and inflammation-linked pathways across retinal diseases, the core focus of this study was on neovascular AMD and DR, where VEGF-driven angiogenesis and anti-VEGF therapy are clinically pertinent. Findings related to GLAU and RP are therefore presented as exploratory, hypothesis-generating data rather than as evidence of VEGF-dependent mechanisms in those conditions.

Our comparative analysis of multiple retinal disorders (AMD, DR, GLAU, and RP) is grounded in the principles of network medicine.^69^ This landmark framework posits that apparently distinct pathophenotypes can be mapped onto the human interactome to reveal shared molecular relationships, including common network modules or pathways, even when clinical presentations and primary therapeutic targets differ. By examining these diseases jointly, shared pathobiological mechanisms—particularly aging- and inflammation-related hubs—can be uncovered.

Unlike prior analyses confined to single-disease genomics, our study's cross-disease integration revealed common aging mechanisms, which we then validated through literature-based contextualization. This dual in silico–experimental approach provides a level of novelty and translational relevance absent from earlier bioinformatics reports.

Utilizing 14 key genes identified by qPCR and prioritized through multilayered bioinformatics analyses, this study establishes a preliminary systems-level map of suboptimal anti-VEGF responses. The 14 genes were literature-selected candidates that our bioinformatics workflow confirmed and contextualized.

Neutralization of VEGF via htsFLT01 extends its influence beyond angiogenesis to encompass broader inflammatory and stress-responsive circuits.^70^^–^^72^ This occurs through direct molecular neighbors such as VTN, HSP90AA1, CTNNB1, KDR, HSPA4, STAT3, and SMAD3. The VEGF–VEGFR2 axis itself is modulated by EGFR and SIRT1, which amplify the suboptimal response to anti-VEGF therapy through mTOR, ERK, and autophagy pathways.^73^ Within RPE clusters, HSP90AA1 and its interactors—MMP2, LYN, FN1, STAT3, and HIF1A—further strengthen this pro-resistance network.

The clustered pathways identified—spanning inflammation, vascular remodeling, survival signaling, adhesion, metabolism, and developmental signaling—act as interlocked drivers of therapy escape.^10^^,^^17^^,^^74^^–^^78^ For example, VTN links PI3K/AKT and Notch pathways, modulating extracellular matrix (ECM) and epithelial-mesenchymal transition (EMT)^79^^,^^80^; HSPA4 and HSP90AA1 drive angiogenic stress responses via ANGPT2 and mTOR^81^^,^^82^; and CTNNB1 supports nuclear β-catenin activation, influencing drug resistance and EMT through WNT and SHH signaling.^83^^,^^84^ Other core mediators include SMAD3 and HIF1A, which fuel EMT and angiogenesis via VEGF, TNF, and MMP9^85^^–^^88^; MMP2, a downstream target of STAT3 and EGFR, reinforces Choroidal neovascularization and ECM remodeling^89^^–^^92^; and SIRT1, through autophagy and metabolic control, regulates cell fate in anti-VEGF refractory RPE environments.^93^^–^^97^ Further, IL-17, TNF, and chemokine signaling disrupt the blood–retinal barrier and promote immune cell recruitment.^98^^–^^101^ Mechanotransduction via integrins, Notch, and fluid shear stress integrates vascular remodeling with resistance.^102^^,^^103^ PI3K–AKT, RAS–MAPK, and AMPK–ULK1 promote bypass survival mechanisms,^104^^,^^105^ while Rap1, ECM remodeling, and cytoskeletal changes sustain angiogenesis.^106^

The identification of five axis-based gene clusters—ANGPT2–MAPK1–DDIT3, IL6–HSPA5–VEGFC–GRB2, FOXO1–STAT3, CCL2–CXCL1–MMP9, and MMP14—reveals early signatures of suboptimal treatment response after htsFLT01 delivery.^107^ These axes converge on known drivers of endoplasmic reticulum (ER) stress (DDIT3),^108^ inflammation (IL6, CXCL1),^109^^,^^110^ survival (STAT3, FOXO1),^111^^,^^112^ and matrix remodeling (MMP14, MMP9),^113^ collectively delineating a molecular landscape implicated in resistance mechanisms. ANGPT2 activation via HSP90AA1–LYN–MAPK3 forms a bypass route, while GRP78 and GRB2 link ER stress to PDGF signaling.^114^^,^^115^

Together, these findings define a well-supported systems-level map of suboptimal anti-VEGF responses in retinal neovascular diseases. Through integrative multigene and pathway analyses, we demonstrate that key signaling axes—namely, VEGF–VEGFR2, EGFR–STAT3, PI3K–AKT–mTOR, and HSP90AA1-mediated autophagy—form convergent regulatory hubs driving angiogenesis, immune activation, and cellular adaptation to stress. These axes are not only central to early pathological remodeling in AMD, DR, GLAU, and RP but also critically shape the emergence of therapeutic refractoriness across distinct retinal disease contexts.

Importantly, the identification of compensatory molecular circuits such as the ANGPT2–MAPK1–DDIT3 and IL6–STAT3 pathways highlights potential therapeutic targets beyond VEGF, capable of sustaining angiogenic signaling under anti-VEGF presence. These adaptive circuits involve key players in ER stress, cytokine signaling, and ECM remodeling, reinforcing the multifactorial nature of treatment failure. By mapping these interactions at the level of RPE clusters, our results provided mechanistic granularity that linked therapeutic failure to cell-type–specific molecular reprogramming.

While our network analysis indicates that aging-related pathways (e.g., the SIRT1–FOXO1 axis and ER stress regulators like GRP78) may be involved in suboptimal response to anti-VEGF therapy, we now present this as a hypothesis rather than a confirmed mechanism. These exploratory findings suggest a possible contribution of senescence and protein-folding stress pathways to anti-VEGF suboptimal responses, but further experimental validation is required.

From a translational standpoint, these findings provide a rationale for considering combination therapies that address both VEGF and compensatory resistance pathways. Simultaneously disrupting central refractory axes—such as SIRT1, EGFR, and MMP9—alongside VEGF inhibition may offer synergistic efficacy, particularly when coupled with precision delivery tools like the MiRGD fusion peptide system.^116^^–^^118^ Our demonstration of time-dependent gene expression changes following htsFLT01 delivery into RPE cells confirms that molecular compensations begin early, underscoring the value of early intervention and combinatorial blockade.

Looking ahead, these findings form the foundation for a broader research program aimed at developing and validating next-generation peptide-based nanodelivery systems targeting interconnected angiogenic and inflammatory pathways. Future in vivo studies will test these rationally designed combinations in preclinical models of AMD and DR, evaluating their ability to prevent neovascularization and mitigate refractory response onset. Concurrently, computational modeling and patient-derived datasets will refine the prioritization of therapeutic targets based on cluster-specific vulnerability and pathway dominance.

While we used the immortalized human RPE cell line (a widely used model in antiangiogenic research chosen for its RPE-like properties and experimental tractability),^119^^,^^120^ we acknowledge that such cells may not fully behave like primary RPE cells in vivo, which is a limitation of this study. Moreover, the isolation and preparation of primary RPE cells from patients with ocular diseases are associated with considerable challenges, including limited tissue availability, ethical constraints, donor variability, and difficulties in maintaining differentiated phenotypes in culture. Future studies may extend these findings to endothelial or coculture models; however, the present work was designed to dissect RPE-intrinsic regulatory mechanisms.

A limitation of this study is that experiments were performed under basal conditions rather than disease-mimicking environments (e.g., hypoxia, hyperglycemia, inflammatory stress). Future studies incorporating disease-relevant stimuli will be required to model how the identified resistance axes (e.g., SIRT1, EGFR/STAT3) are modulated within a pathological microenvironment and directly test pathological VEGF resistance mechanisms. Additionally, although combining interventions on these convergent hubs could hypothetically enhance antiangiogenic efficacy, this concept remains hypothetical and unproven at this stage. We underscore that additional animal studies or other in vivo evidence are needed to support such a therapeutic strategy.

Ultimately, the study's integrated approach bridges high-dimensional systems biology with experimental validation, offering both conceptual and methodological advances. These findings identify baseline regulatory pathways that may contribute to VEGF resistance mechanisms when dysregulated under pathological retinal conditions. By illuminating shared disease-driving axes and resistance mechanisms across four major blinding disorders (AMD, DR, GLAU, and RP), the study provides a unified framework. This framework is crucial for informing several key areas, including biomarker discovery, therapeutic design, and long-term vision preservation strategies. Notably, we translated our computational predictions into an experimental context via nanoparticle delivery of the htsFLT01 gene, confirming its functional impact in an hRPE cell-based model. This step elevates our study from a purely computational exercise to one with tangible translational evidence, distinguishing it from previous dataset-based studies.

## Supplementary Material

Supplement 1

Supplement 2

Supplement 3

Supplement 4

Supplement 5

Supplement 6

Supplement 7

Supplement 8

Supplement 9

Supplement 10

Supplement 11

Supplement 12

Supplement 13

Supplement 14

Supplement 15

Supplement 16

Supplement 17

Supplement 18

Supplement 19

Supplement 20

Supplement 21

Supplement 22

Supplement 23

Supplement 24

Supplement 25

Supplement 26

Supplement 27

Supplement 28

Supplement 29

Supplement 30

Supplement 31

Supplement 32

Supplement 33

Supplement 34

Supplement 35

Supplement 36

Supplement 37

Supplement 38

Supplement 39

Supplement 40
