# Supplementary material for: Suboptimal Responses to Anti-VEGF in Retinal Neurovascular Diseases: Linking Aging and Alternative Angioinflammatory Pathways
Source: Invest Ophthalmol Vis Sci. 2026 May 4;67(5):4. doi: 10.1167/iovs.67.5.4 (PMC13164572; doi:10.1167/iovs.67.5.4)
Supplement: Supplement 1 [file iovs-67-5-4_s001.docx]

**
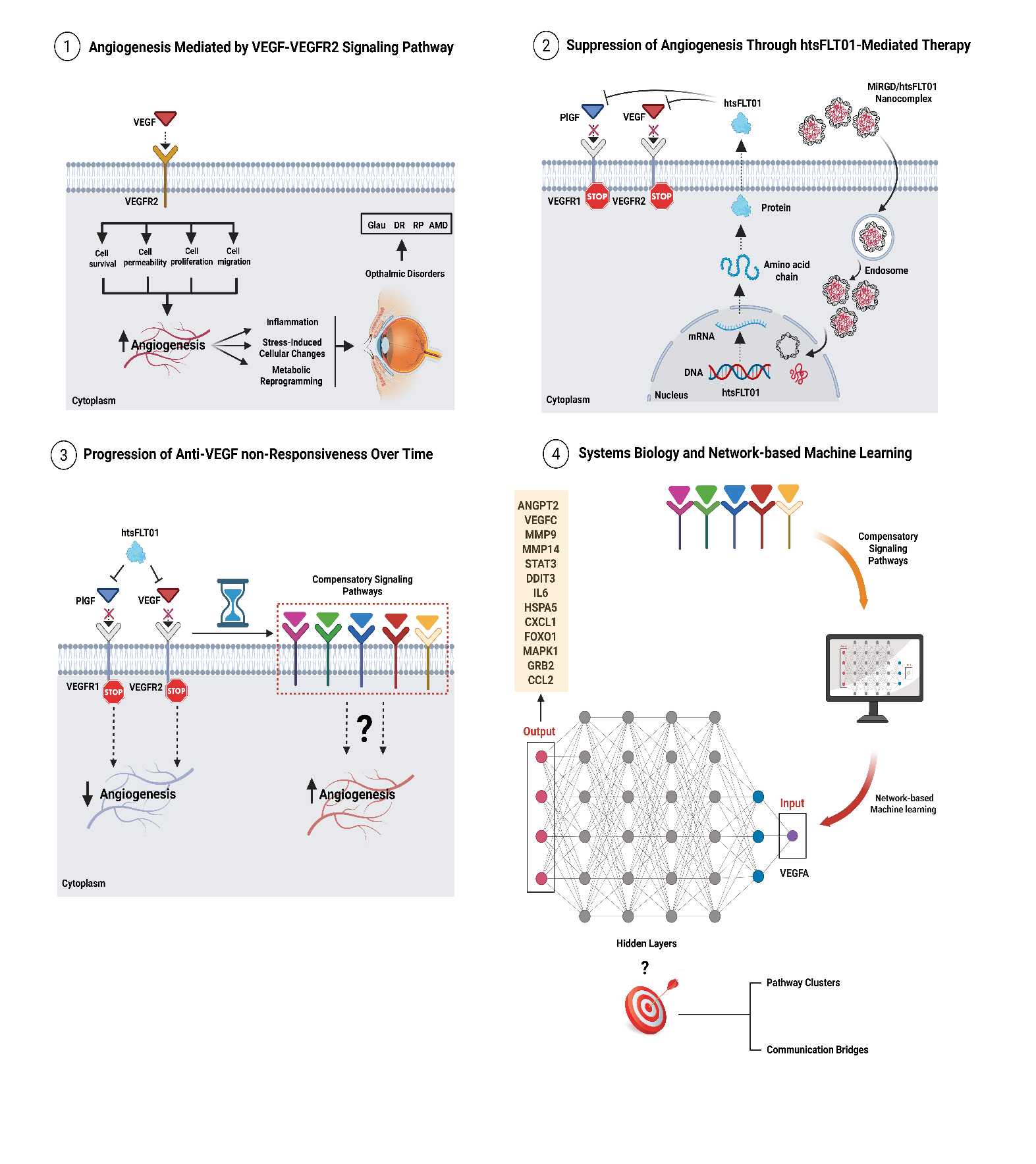
**

**Scheme 1.**

**Panel 1: Angiogenesis Mediated through VEGF/VEGFR2 Signaling Pathway.** When VEGF binds to its receptor VEGFR2, it triggers signaling pathways that promote cell migration, proliferation, and increased vascular permeability; key steps in angiogenesis. However, when combined with inflammation, oxidative stress-induced cellular changes, and abnormal metabolic alterations, the VEGF-VEGFR2 pathway can contribute to the development of serious ophthalmic disorders, including AMD, DR, RP, and GLAU.

**Panel 2: Mitigation of Angiogenesis Through htsFLT01-Mediated Therapy.** The htsFLT01/MiRGD nanocomplex, via MiRGD peptide motifs, attaches to cells, escapes from the endosome, and is transported to the nucleus. htsFLT01 is then expressed and secreted, binding to the PlGF and VEGF. This binding competitively inhibits ligand-receptor interactions with VEGFR1 and VEGFR2. Ultimately, pro-angiogenic signaling pathways are effectively suppressed.

**Panel 3: Anti-VEGF Suboptimal Response.** Despite the considerable antiangiogenic efficacy of htsFLT01 and other agents that block the primary VEGF-VEGFR2 axis, a potential for suboptimal response to these treatments exists. This occurs due to compensatory and/or compromised anti-angiogenic and anti-inflammatory pathways that facilitate the development of new vasculature despite the blockage of the implicated primary pathway. Examining the function of these compensatory and/or compromised anti-angiogenic and anti-inflammatory pathways in the emergence of anti-VEGF suboptimal response is a matter that requires attention.

**Panel 4: Systems Biology and Network-based Machine Learning.** The examination of the hidden layers in the information transfer from VEGF (input data) to output data within compensatory and/or compromised anti-angiogenic and anti-inflammatory pathways resulted in the identification of a black box that may contribute to the development of anti-VEGF suboptimal response involving pathway clusters and communication bridges.

The schematic was designed by BioRender (https:// BioRender.com/q2kawlt.(


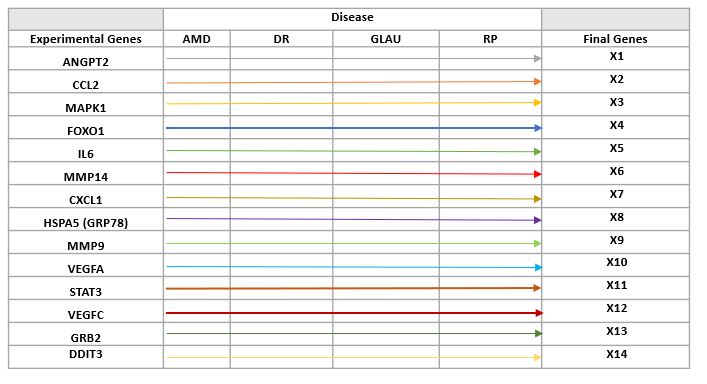


**Supplementary Figure S2**


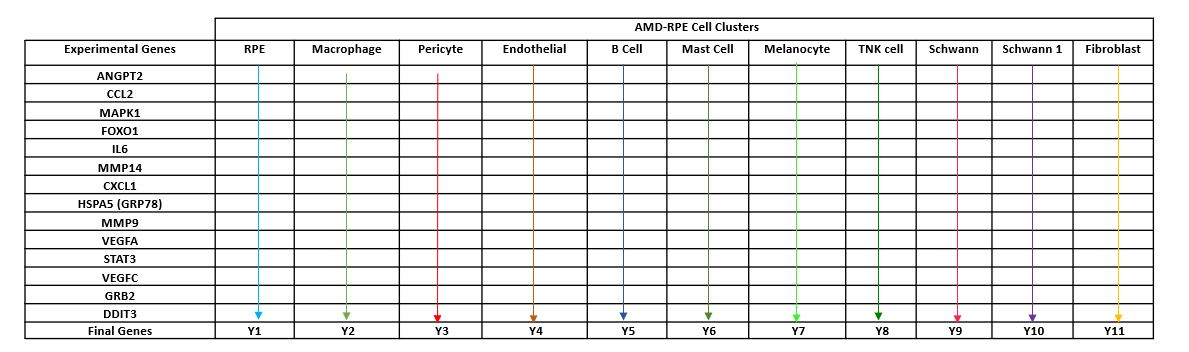


**Supplementary Figure S3**


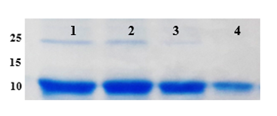


**Supplementary Figure S4.** The purified peptide bands were stained with Coomassie Blue in 15% Tris-Glycine SDS-PAGE. Lanes 1-4: Different elutions of the MiRGD peptide. The calculated molecular weight of MiRGD is 9.6 kDa.

**
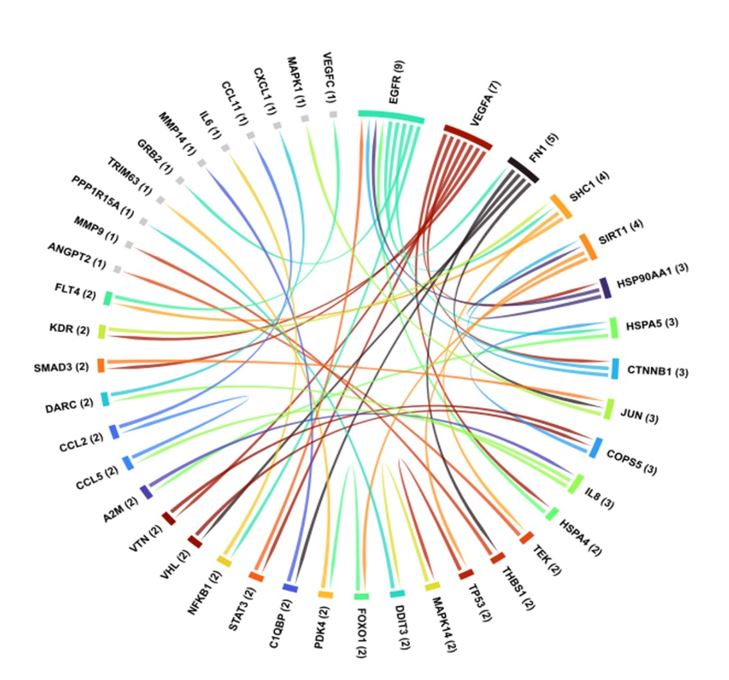
**

**Supplementary Figure S5. Visualization of VEGF–gene interaction networks through Chord diagram.**
The Chord diagram depicts the interrelationships among VEGF-associated genes, illustrating shared connections and central hubs extracted from disease-specific datasets. The circular layout displays genes around the perimeter, annotated with their symbols and interaction degree (in parentheses). VEGFA exhibits the highest connectivity (degree 7), underscoring its pivotal role in vascular and cellular response pathways. The chord diagram offers valuable complementary insights into the distribution of VEGF signaling across diverse downstream target pathways, highlighting key regulatory hubs with potential relevance to both physiological and pathological angiogenesis as well as strategic opportunities for therapeutic modulation.


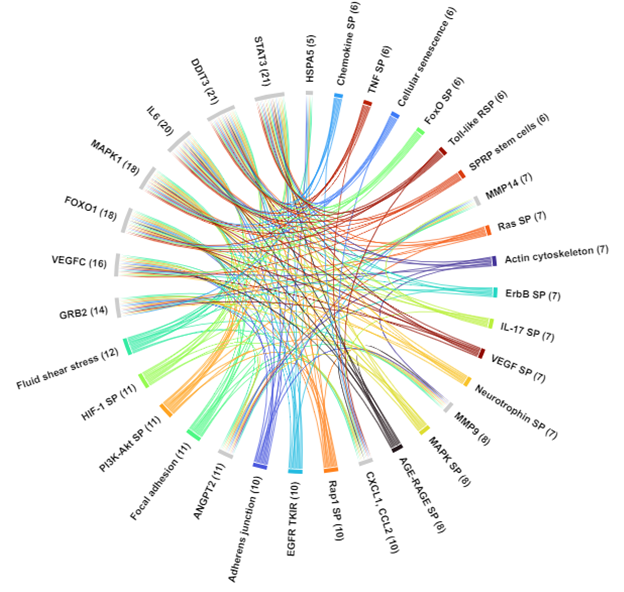


**Supplementary Figure S6. Chord diagram mapping gene–pathway interactions underlying VEGF signaling**. The chord diagram visualizes the connectivity between key VEGF-associated genes and enriched signaling pathways. Major hub genes (e.g., STAT3, MAPK1/ERK2, FOXO1, IL6, VEGFC) exhibit broad connectivity across multiple pathways, including PI3K–Akt, HIF-1, EGFR tyrosine kinase inhibitor resistance (TKIR), AGE–RAGE, Toll-like receptor, Rap1, TNF, chemokine, and focal adhesion signaling. These extensive overlaps illustrate prominent Chord diagram of gene–pathway interactions associated with VEGF cross-talk and shared regulatory nodes sustaining angiogenic programs. Gene labels show the total number of connected pathways in parentheses. Abbreviations: SP = signaling pathway; TKIR = tyrosine kinase inhibitor resistance; RSP = receptor signaling pathway; SPRP = signaling pathway regulating pluripotency.


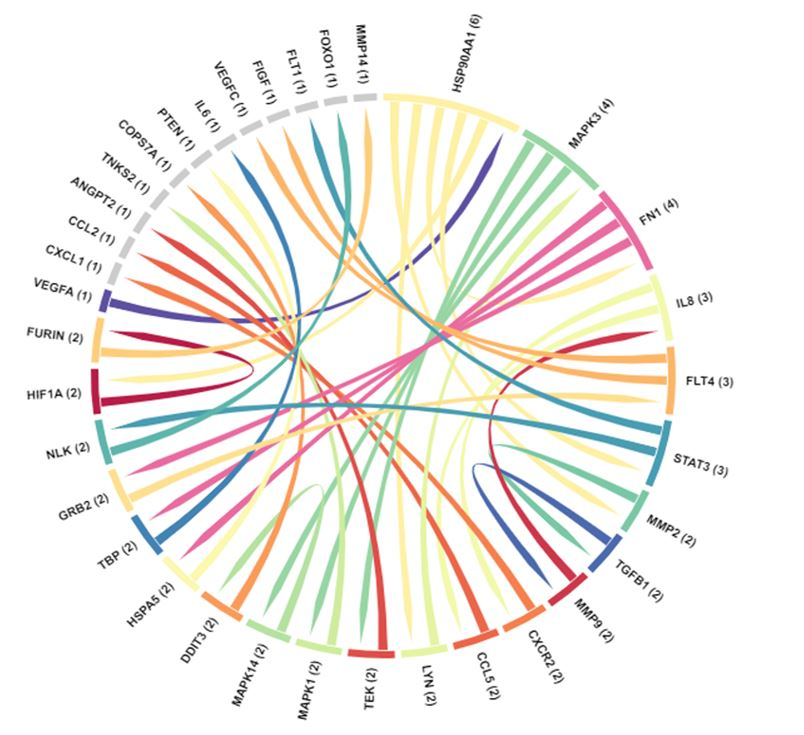


**Supplementary Figure S7. Chord diagram illustrating VEGFA-centric gene interaction network in the RPE cluster.** The chord diagram depicts directed interactions between VEGFA (central hub) and associated genes in the RPE cluster. Numbers in parentheses next to gene names indicate the degree of connectivity (number of links). Key hub genes with extensive cross-linking—such as HSP90AA1 (6 links), MAPK3 (4), FN1 (4), IL8 (3), FLT4 (3), and STAT3 (3)—highlight central nodes in angiogenic, inflammatory, and signaling pathways within the RPE microenvironment. These visualizations highlight the interconnected regulatory axes that sustain VEGF-driven processes within the RPE microenvironment.


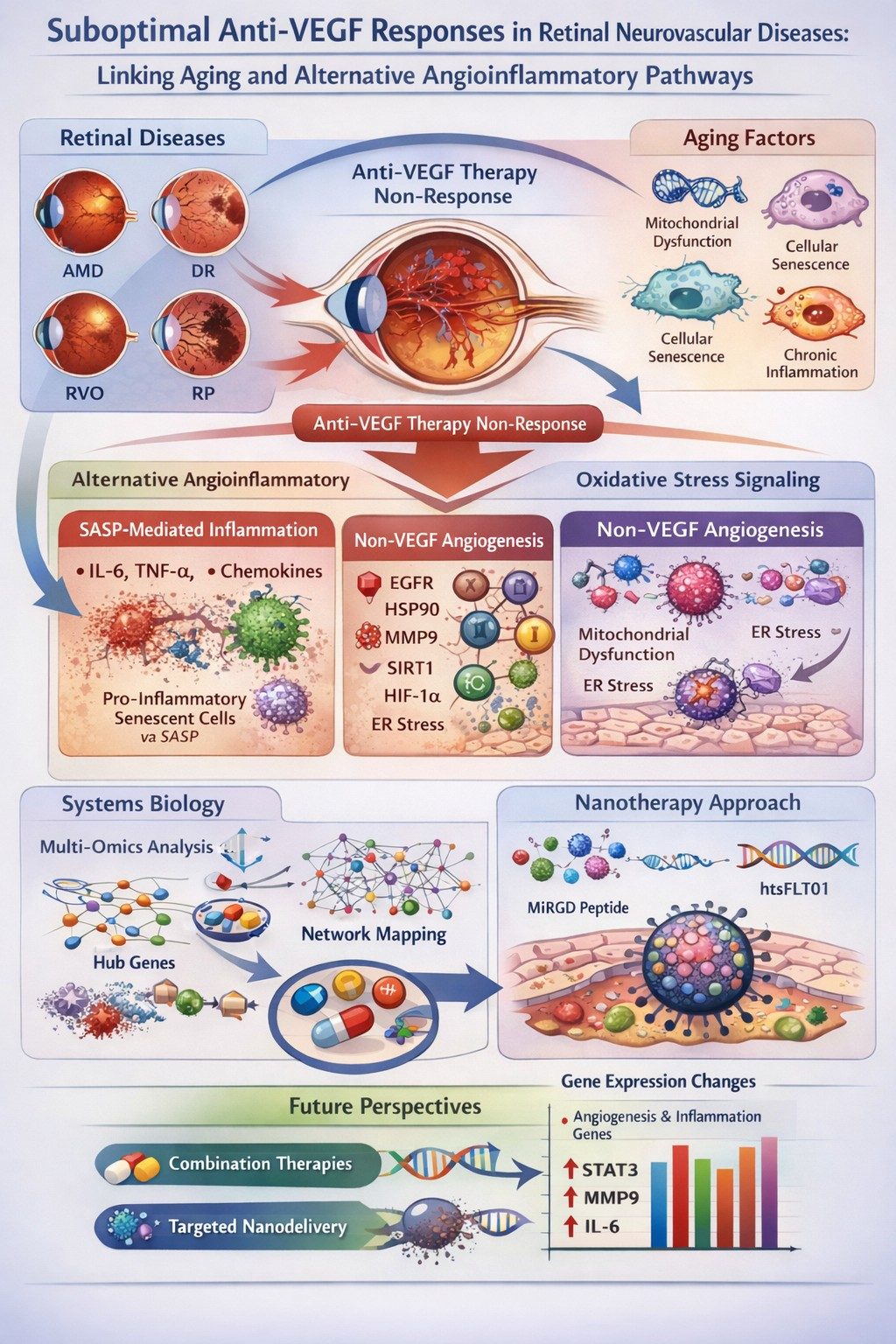


Some eye diseases don’t improve with common anti-VEGF treatments. As we age, cell stress, inflammation, and other growth signals can keep damaging blood vessels in the retina. By studying these pathways and using smart nanotherapy and combination treatments, scientists hope to create better, longer-lasting vision care.
