# Supplementary material for: Suboptimal Responses to Anti-VEGF in Retinal Neurovascular Diseases: Linking Aging and Alternative Angioinflammatory Pathways
Source: Invest Ophthalmol Vis Sci. 2026 May 4;67(5):4. doi: 10.1167/iovs.67.5.4 (PMC13164572; doi:10.1167/iovs.67.5.4)
Supplement: Supplement 40 [file iovs-67-5-4_s040.docx]

**Supplementary Table S39.** The relative expression of candidate genes in human RPE cells transfected with the htsFLT01/MiRGD samples measured at two different time points.

| **Gene Name** | **Relative Expression Level** | | | |
| --- | --- | --- | --- | --- |
|  | **48 h Post Transfection** | | **72 h Post Transfection** | |
|  | **Fold Change** | **P-value** | **Fold Change** | **P-value** |
| **ANGPT2** | **14.96649** | **0.0010** | **2.189897** | **0.0419** |
| **CCL2** | **42.63702** | **0.0115** | **9.851142** | **0.0434** |
| **MAPK** | **1.661286** | **0.0054** | **1.375618** | **0.0222** |
| **FOXO1** | **2.432674** | **0.0286** | **2.006841** | **0.0267** |
| **IL6** | **2.523982** | **0.0027** | **1.224819** | **-** |
| **MMP14** | **5.972868** | **0.0493** | **2.205951** | **0.0392** |
| **CXCL1** | **3.402941** | **0.0183** | **4.402741** | **0.0251** |
| **GRP78** | **2.084745** | **0.0426** | **1.003094** | **-** |
| **MMP9** | **9.778266** | **0.0078** | **4.82843** | **0.0302** |
| **VEGFA** | **2.584421** | **0.0004** | **2.049483** | **0.0286** |
| **STAT3** | **2.911445** | **0.0022** | **1.314784** | **0.0020** |
| **VEGFC** | **2.808456** | **0.0383** | **1.959264** | **0.0018** |
| **GRB2** | **3.176988** | **0.0252** | **1.411679** | **0.0230** |
